# Supplementary material for: Remote tuning of single-atom Fe-N5 sites via high-coordination defects for enhanced Fenton-like water decontamination
Source: Nat Commun. 2025 Nov 25;16:10455. doi: 10.1038/s41467-025-65425-4 (PMC12647131; doi:10.1038/s41467-025-65425-4)
Supplement: Supplementary file 1 — Supplementary Information [file 41467_2025_65425_MOESM1_ESM.pdf]

Supplementary Information

Remote Tuning of Single-Atom Fe-N_5 Sites via High-Coordination Defects for Enhanced Fenton-Like Water Decontamination

Sijia Jin[1,2], Wenxian Tan[2], Yilin Huang[3], Yi Wang[2], Zhiqiao He[2], Haiyan Zhang[1], Shuang Song[2], Yaqi Cai[1,4] and Tao Zeng[1,2,5]*

[1]Zhejiang Key Laboratory of Environment and Health of New Pollutants, School of Environment, Hangzhou Institute for Advanced Study, University of Chinese Academy of Sciences, Hangzhou, 310024, P.R. China.

[2] Zhejiang Key Laboratory of Low-carbon Control Technology for Industrial Pollution, College of Environment, Zhejiang University of Technology, Hangzhou, Zhejiang, 310032, P.R. China.

[3]Office for Environmental Programs, Faculty of Science, The University of Melbourne, Parkville, Victoria 3010, Australia.

[4]State Key Laboratory of Environmental Chemistry and Ecotoxicology, Research Center for Eco-Environmental Sciences, Chinese Academy of Sciences, Beijing, 100085, P.R. China.

[5]Shaoxing Research Institute, Zhejiang University of Technology, Shaoxing, 312000, P. R. China.

*** Corresponding Author**

Tao Zeng. Email: zengtao@ucas.ac.cn; Tel: +86-571-88320726.

Supplementary Figures

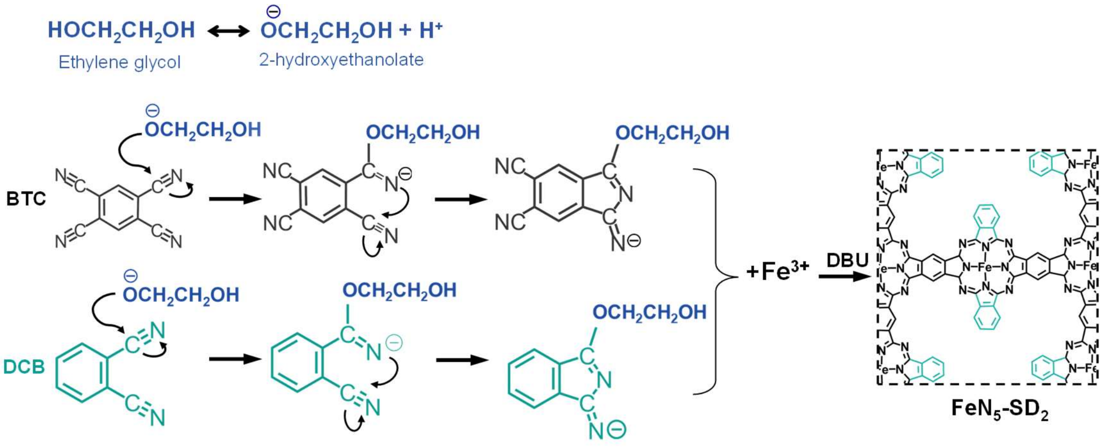

Supplementary Fig. 1 | Synthetic scheme of as-prepared FeN_5 SACs. Formation procedure of FeN_5-SD_2 using 1,2-dicyanobenzene (DCB), and benzene-1,2,4,5-tetracarbonitrile (BTC) as precursors.

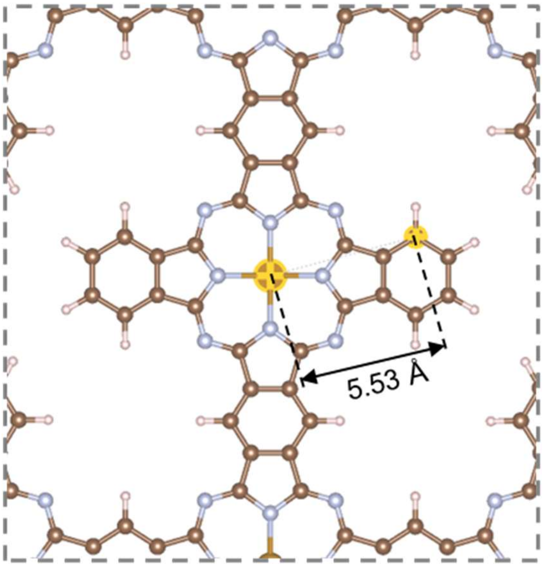

Supplementary Fig. 2 | The spatial range of remote modulation in catalyst structures. Spatial distance from defect sites to Fe centers in FeN_5-SD_2. Crystal structures visualized using VESTA software[1].

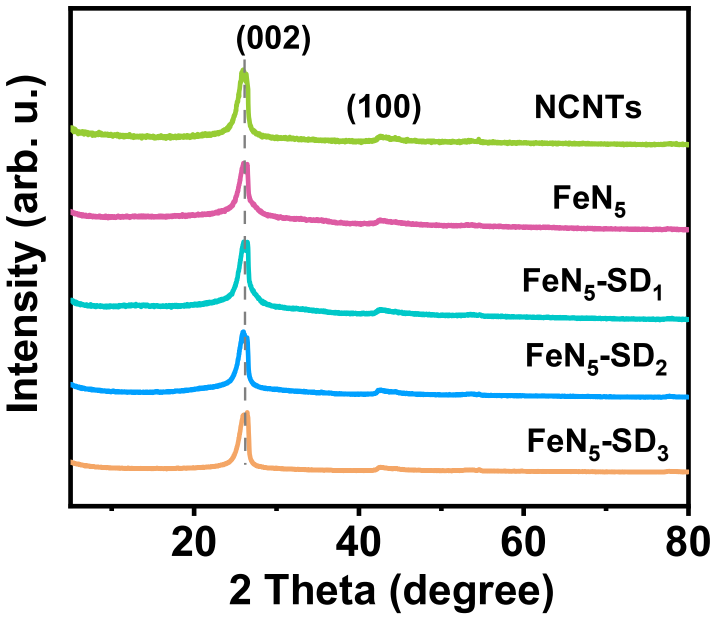

Supplementary Fig. 3 | XRD characterization results. XRD spectra of FeN_5, FeN_5-SD_1, FeN_5-SD_2, FeN_5-SD_3 and NCNTs. Source data are provided as a Source Data file.

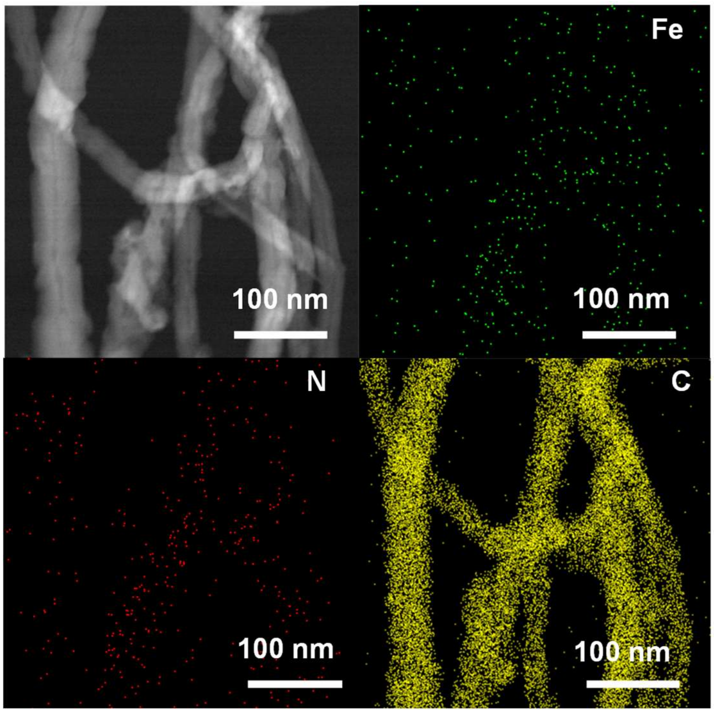

Supplementary Fig. 4 | TEM characterization results of FeN_5-SD_2. TEM element mapping (Fe, N, C) images of FeN_5-SD_2.

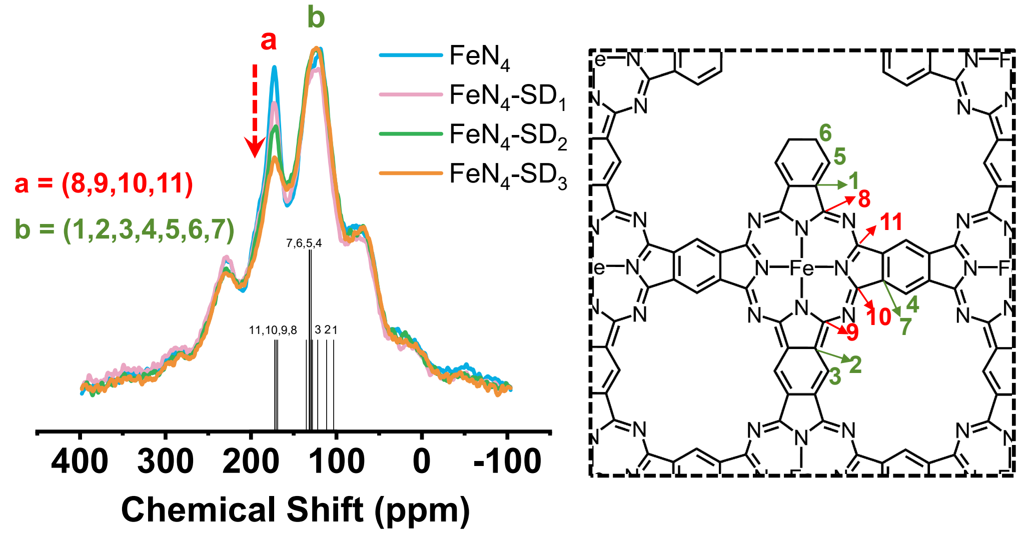

Supplementary Fig. 5 | Solid-state ^{13}C NMR characterization results. Solid-state ^{13}C NMR spectra and ChemDraw 20.0 "predicted" of FeN_4, FeN_4-SD_1, FeN_4-SD_2 and FeN_4-SD_3. Source data are provided as a Source Data file.

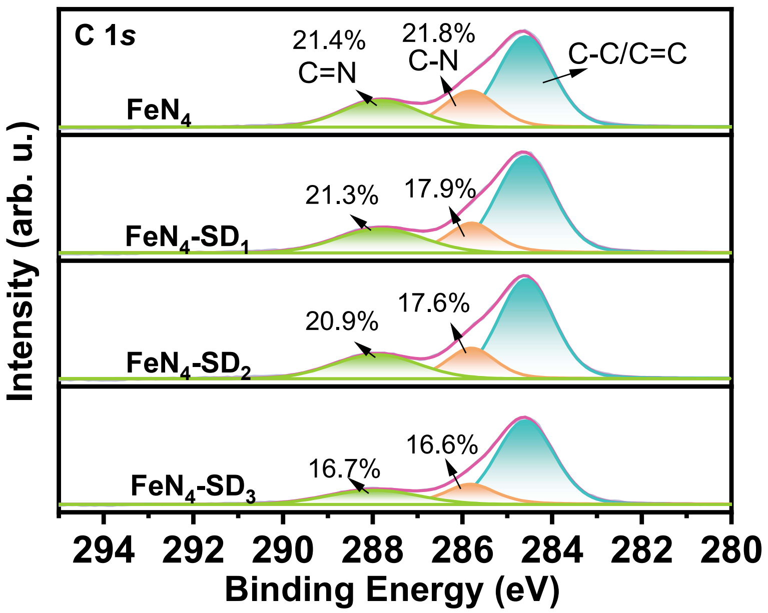

Supplementary Fig. 6 | XPS characterization results. XPS C 1*s* spectra of FeN_4, FeN_4-SD_1, FeN_4-SD_2 and FeN_4-SD_3. Source data are provided as a Source Data file.

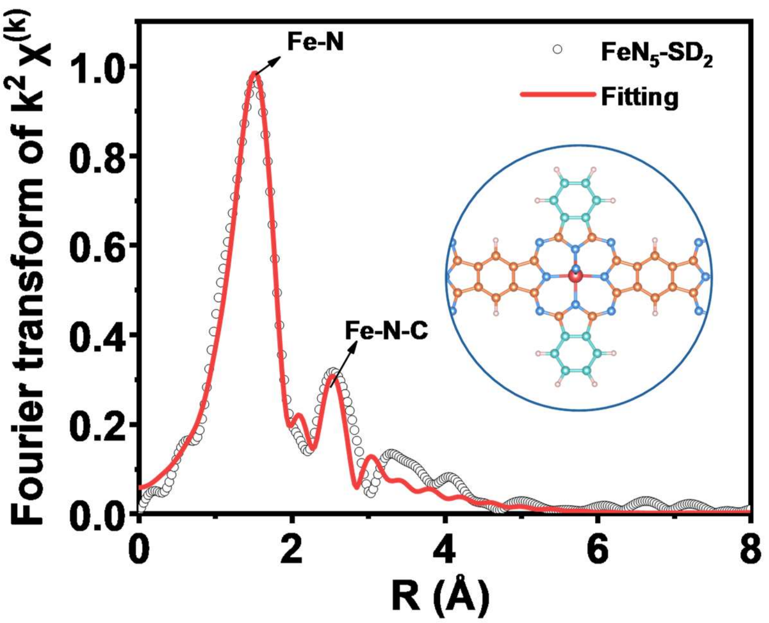

Supplementary Fig. 7 | EXAFS curve fitting results. The corresponding EXAFS fitting curves for FeN_5-SD_2 and the structural model of FeN_5-SD_2. Crystal structures visualized using VESTA software[1]. Source data are provided as a Source Data file.

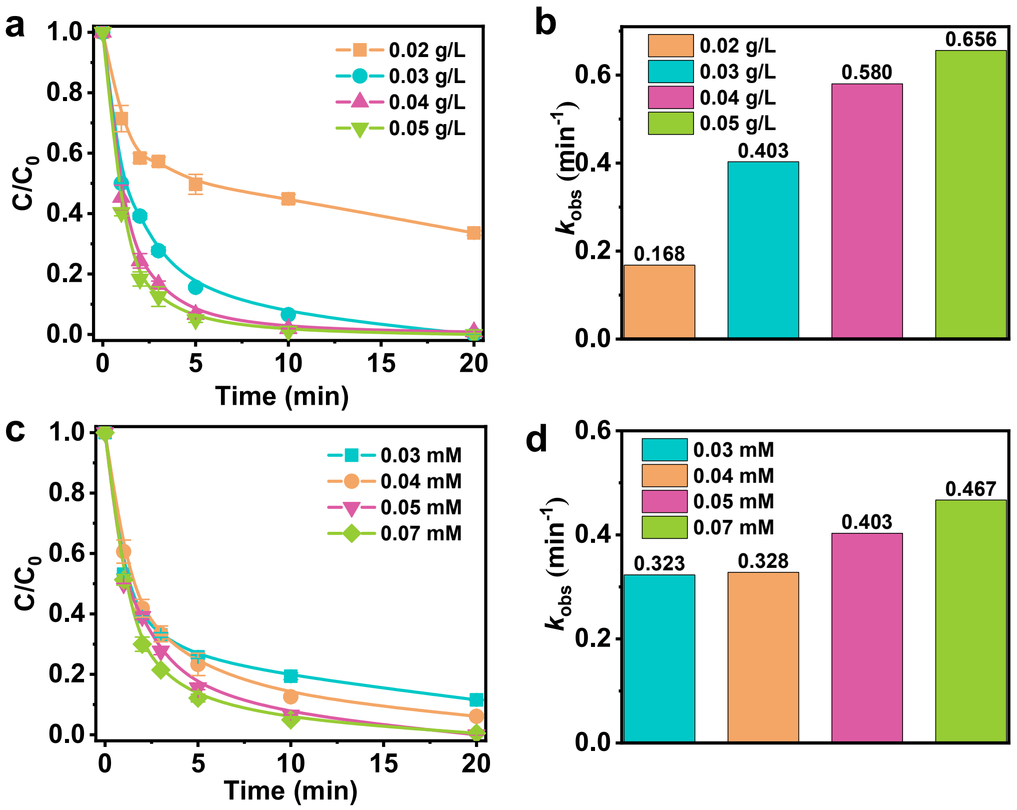

Supplementary Fig. 8 | Effect of different catalyst dosage and PMS concentrations on degradation performance. BPA degradation and the corresponding k_{obs} in FeN_5-SD_2/PMS system under different conditions: **a-b** catalyst dosage and **c-d** PMS concentration. Error bars indicate standard deviation derived from three parallel measurements. Routine conditions: [BPA] = 80 μM, [catalyst] = 0.03 g L^{-1}, [PMS] = 0.05 mM, temperature = 25 °C, without pH adjustment. Source data are provided as a Source Data file.

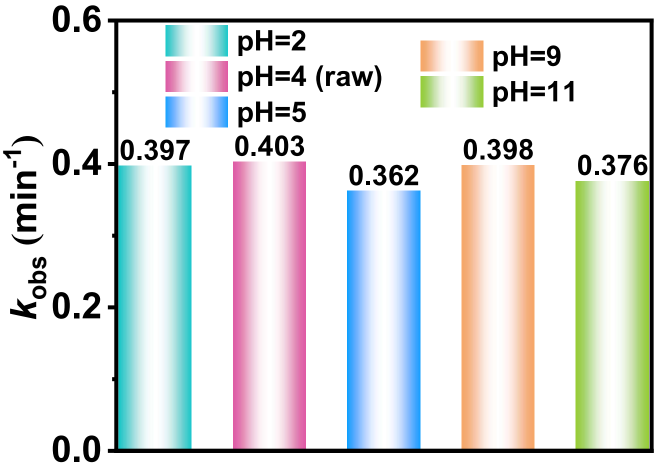

Supplementary Fig. 9 | Effect of different pH on degradation performance. The corresponding k_{obs} in FeN_5-SD_2/PMS system under different pH. Source data are provided as a Source Data file.

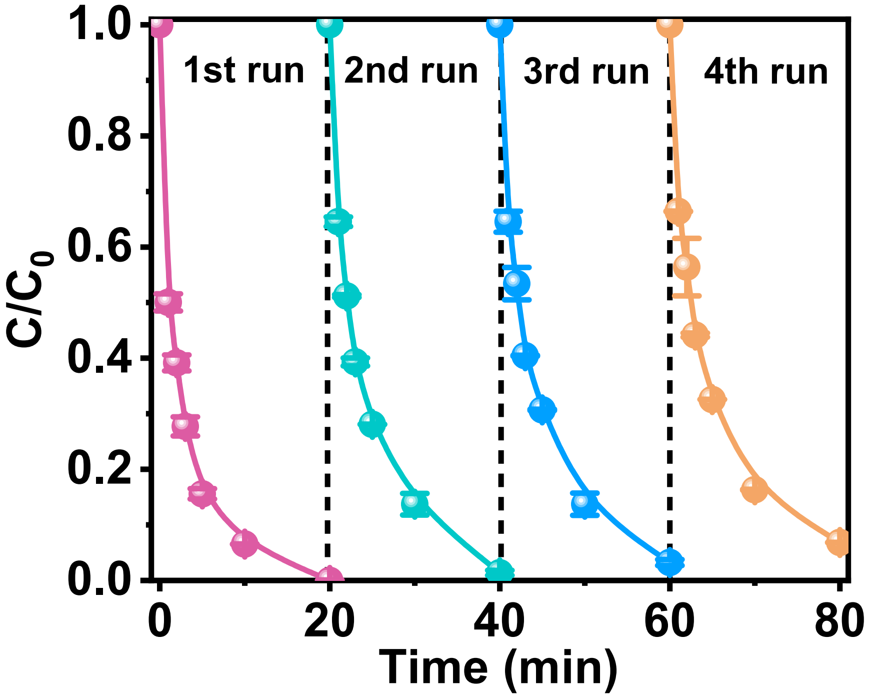

Supplementary Fig. 10 | Cyclic stability test for BPA degradation. Recyclability of FeN_5-SD_2/PMS system for BPA removal. Error bars indicate standard deviation derived from three parallel measurements. Routine conditions: [BPA] = 80 μM, [catalyst] = 0.03 g L^{-1}, [PMS] = 0.05 mM, temperature = 25 °C, without pH adjustment. Source data are provided as a Source Data file.

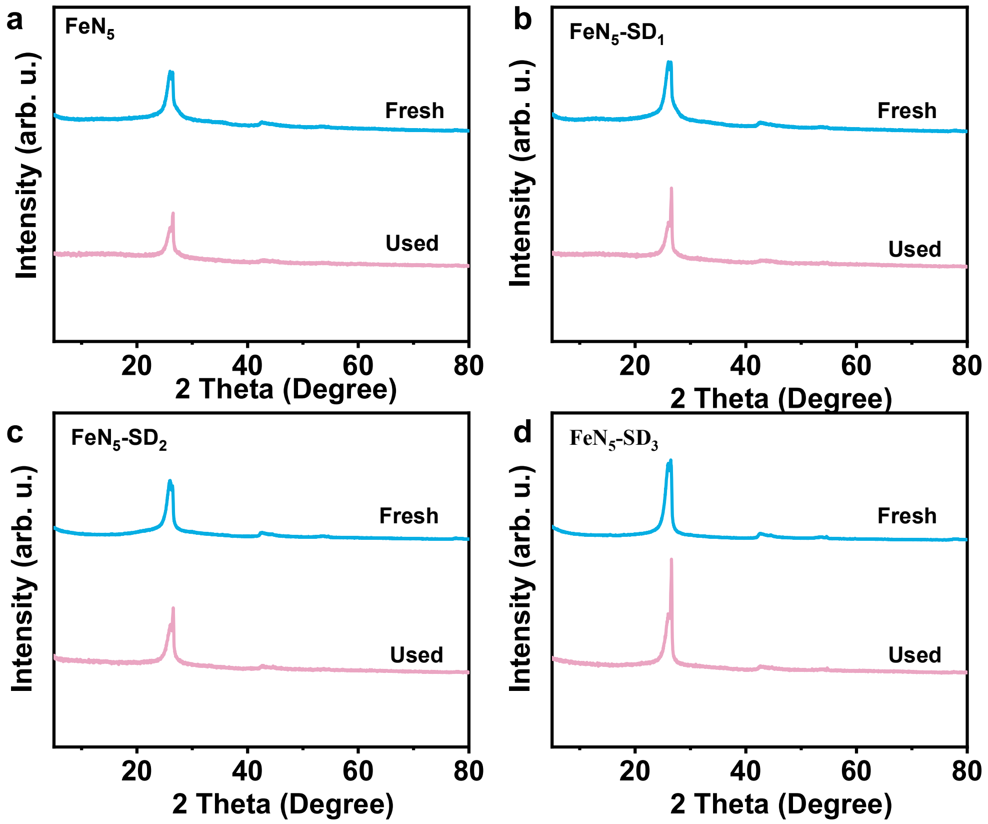

Supplementary Fig. 11 | XRD characterization results. XRD spectra of **a** FeN_5, **b** FeN_5-SD_1, **c** FeN_5-SD_2, and **d** FeN_5-SD_3 before and after use. Source data are provided as a Source Data file.

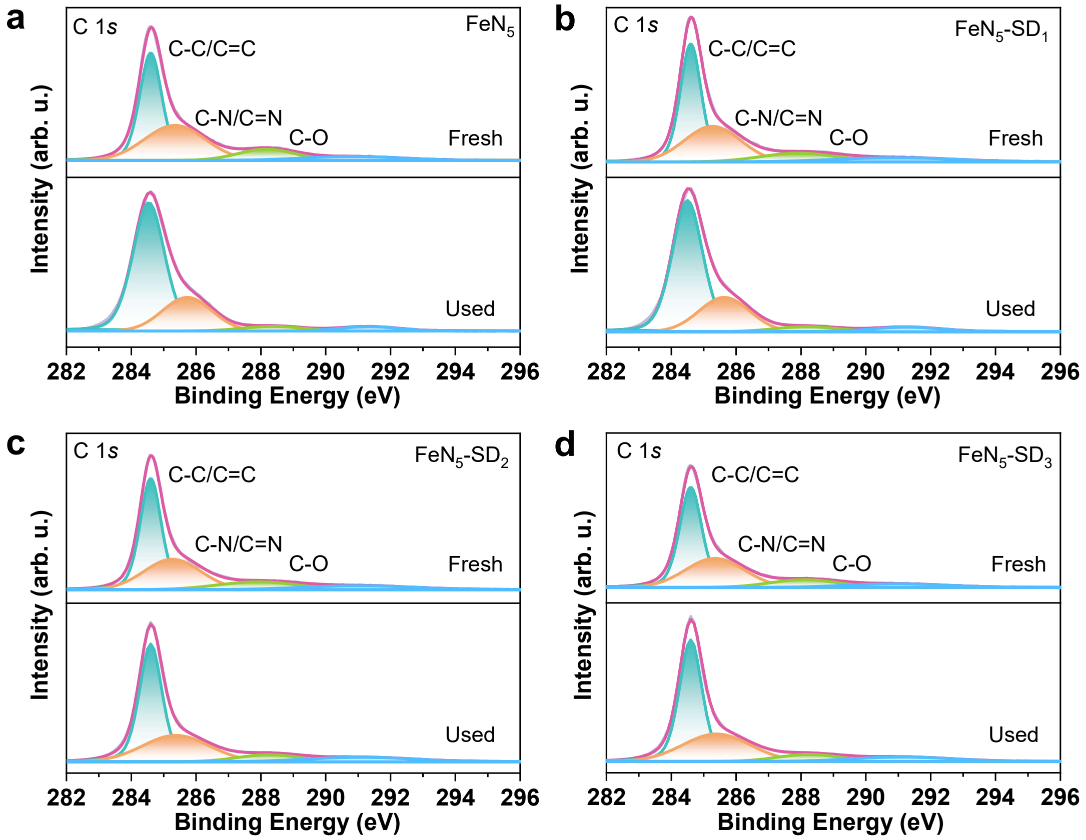

Supplementary Fig. 12 | XPS characterization results. XPS C 1*s* spectra of **a** FeN_5, **b** FeN_5-SD_1, **c** FeN_5-SD_2, and **d** FeN_5-SD_3 before and after use. Source data are provided as a Source Data file.

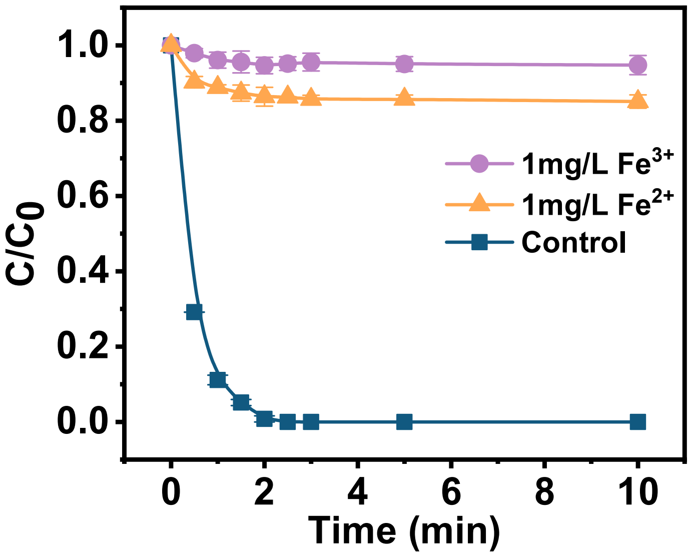

Supplementary Fig. 13 | Effect of homogeneous reaction on BPA degradation. BPA degradation by Fe^{2+}, Fe^{3+} and FeN_5-SD_2 activated PMS system. Error bars indicate standard deviation derived from three parallel measurements. Routine conditions: [BPA] = 80 μM, [PMS] = 0.05 mM, temperature = 25 °C, without pH adjustment. Source data are provided as a Source Data file.

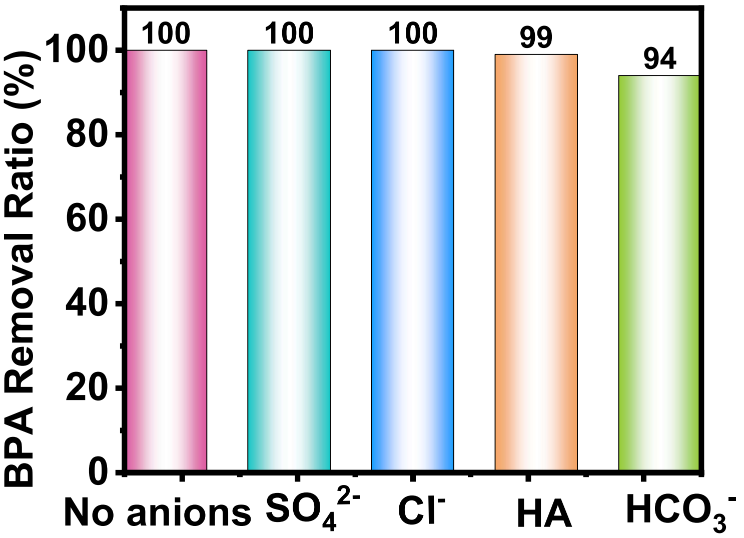

Supplementary Fig. 14 | Effect of different inorganic anions on degradation performance. The BPA removal ratio in FeN_5-SD_2/PMS system under different inorganic anions and HA.

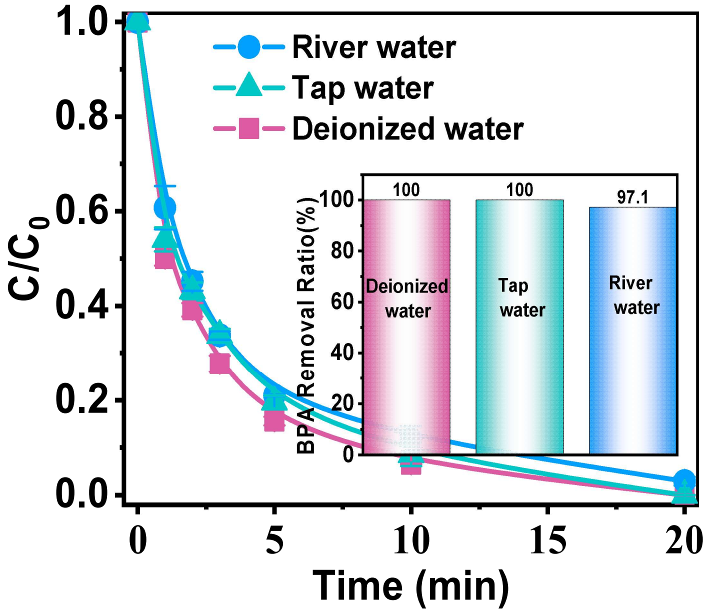

Supplementary Fig. 15 | Effect of different water matrices on degradation performance. BPA degradation in FeN_5-SD_2/PMS system under different water matrices. Error bars indicate standard deviation derived from three parallel measurements. Routine conditions: [BPA] = 80 μM, [catalyst] = 0.03 g L^{-1}, [PMS] = 0.05 mM, temperature = 25 °C, without pH adjustment. Source data are provided as a Source Data file.

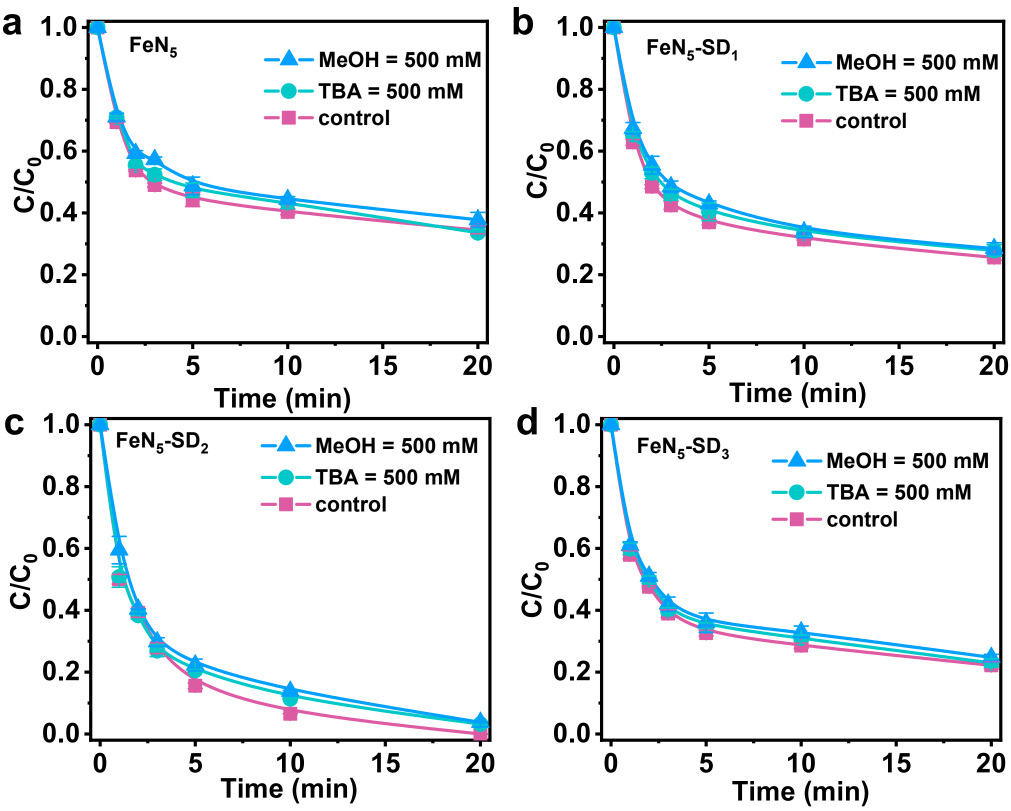

Supplementary Fig. 16 | Investigation of active species. BPA degradation in the **a** FeN_5/PMS, **b** FeN_5-SD_1/PMS, **c** FeN_5-SD_2/PMS, and **d** FeN_5-SD_3/PMS system with MeOH and TBA scavengers. Error bars indicate standard deviation derived from three parallel measurements. Routine conditions: [BPA] = 80 μM, [catalyst] = 0.03 g L^{-1}, [PMS] = 0.05 mM, temperature = 25 °C, without pH adjustment. Source data are provided as a Source Data file.

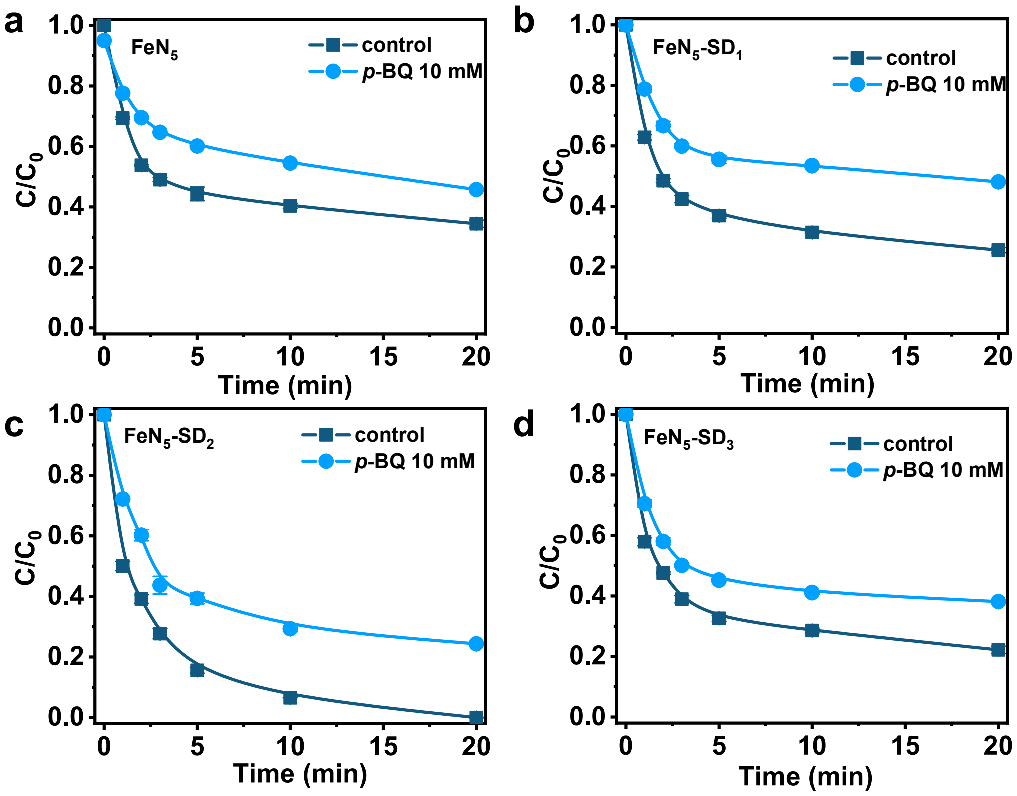

Supplementary Fig. 17 | Investigation of active species. The effect of *p*-BQ on BPA degradation in the **a** FeN_5/PMS, **b** FeN_5-SD_1/PMS, **c** FeN_5-SD_2/PMS, and **d** FeN_5-SD_3/PMS system. Error bars indicate standard deviation derived from three parallel measurements. Routine conditions: [BPA] = 80 μM, [catalyst] = 0.03 g L^{-1}, [PMS] = 0.05 mM, temperature = 25 °C, without pH adjustment. Source data are provided as a Source Data file.

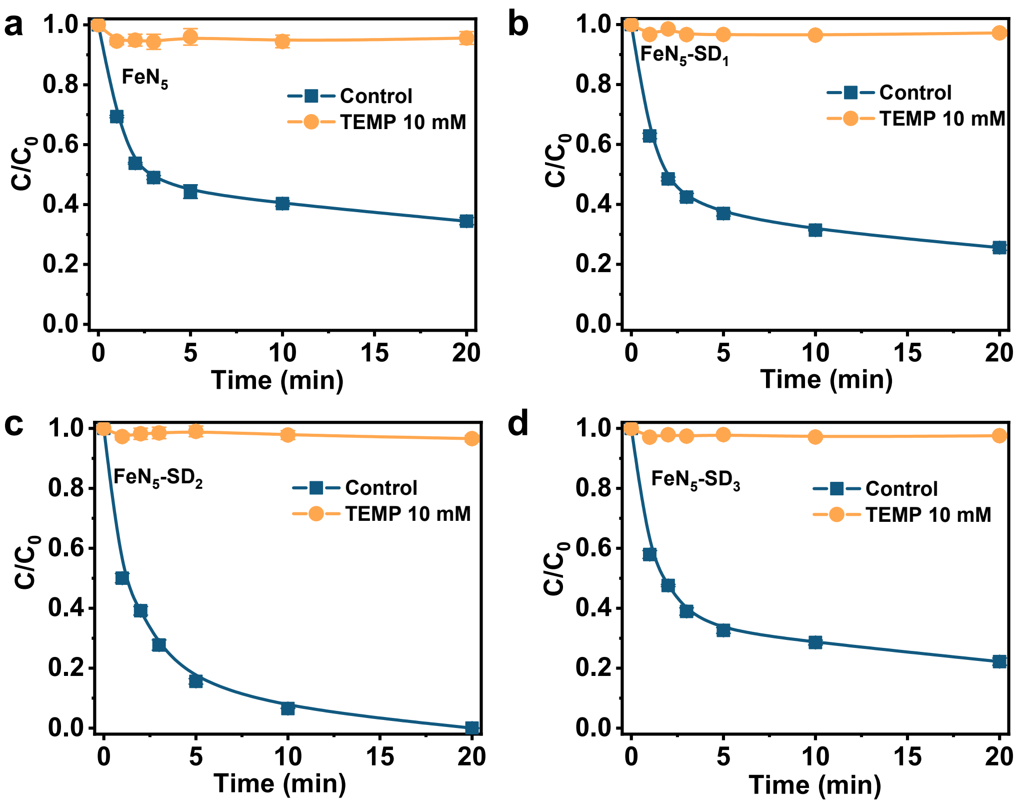

Supplementary Fig. 18 | Investigation of active species. The effect of TEMP on BPA degradation in the **a** FeN_5/PMS, **b** FeN_5-SD_1/PMS, **c** FeN_5-SD_2/PMS, and **d** FeN_5-SD_3/PMS system. Error bars indicate standard deviation derived from three parallel measurements. Routine conditions: [BPA] = 80 μM, [catalyst] = 0.03 g L^{-1}, [PMS] = 0.05 mM, temperature = 25 °C, without pH adjustment. Source data are provided as a Source Data file.

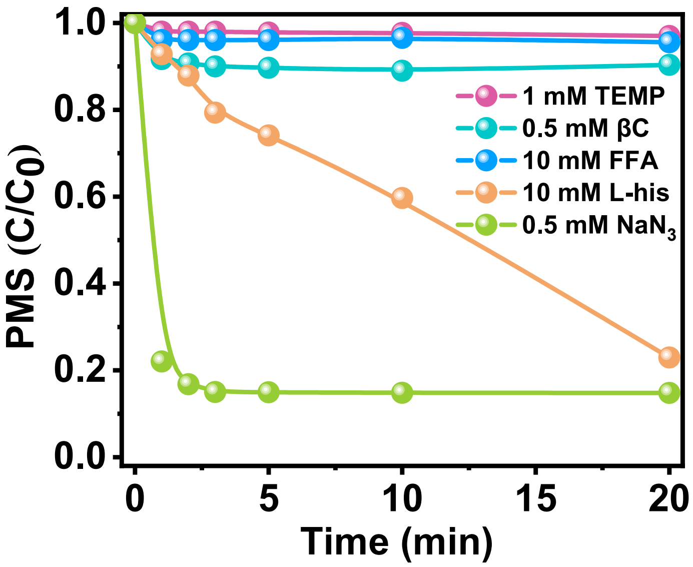

Supplementary Fig. 19 | Effect of different quenchers on PMS consumption. Decomposition rate of PMS with different quenchers. Routine conditions: [PMS] = 0.05 mM, temperature = 25 °C, without pH adjustment. Source data are provided as a Source Data file.

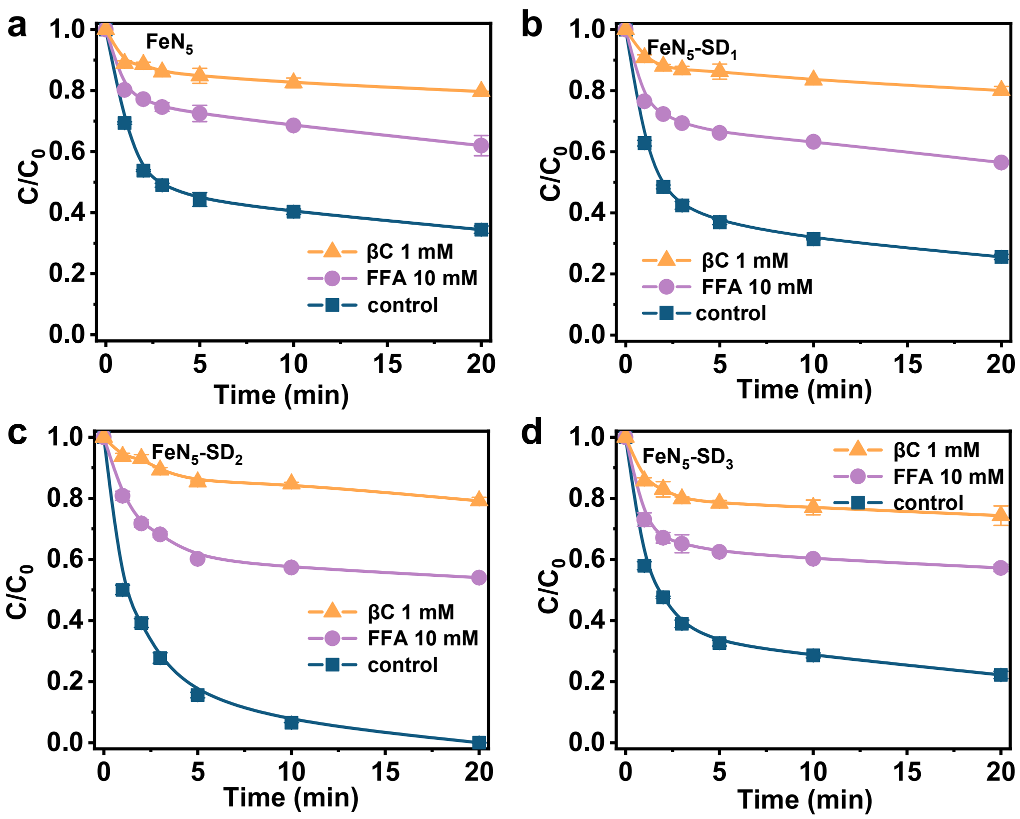

Supplementary Fig. 20 | Investigation of active species. The effect of FFA and βC on BPA degradation in the **a** FeN_5/PMS, **b** FeN_5-SD_1/PMS, **c** FeN_5-SD_2/PMS, and **d** FeN_5-SD_3/PMS system. Error bars indicate standard deviation derived from three parallel measurements. Routine conditions: [BPA] = 80 μM, [catalyst] = 0.03 g L^{-1}, [PMS] = 0.05 mM, temperature = 25 °C, without pH adjustment. Source data are provided as a Source Data file.

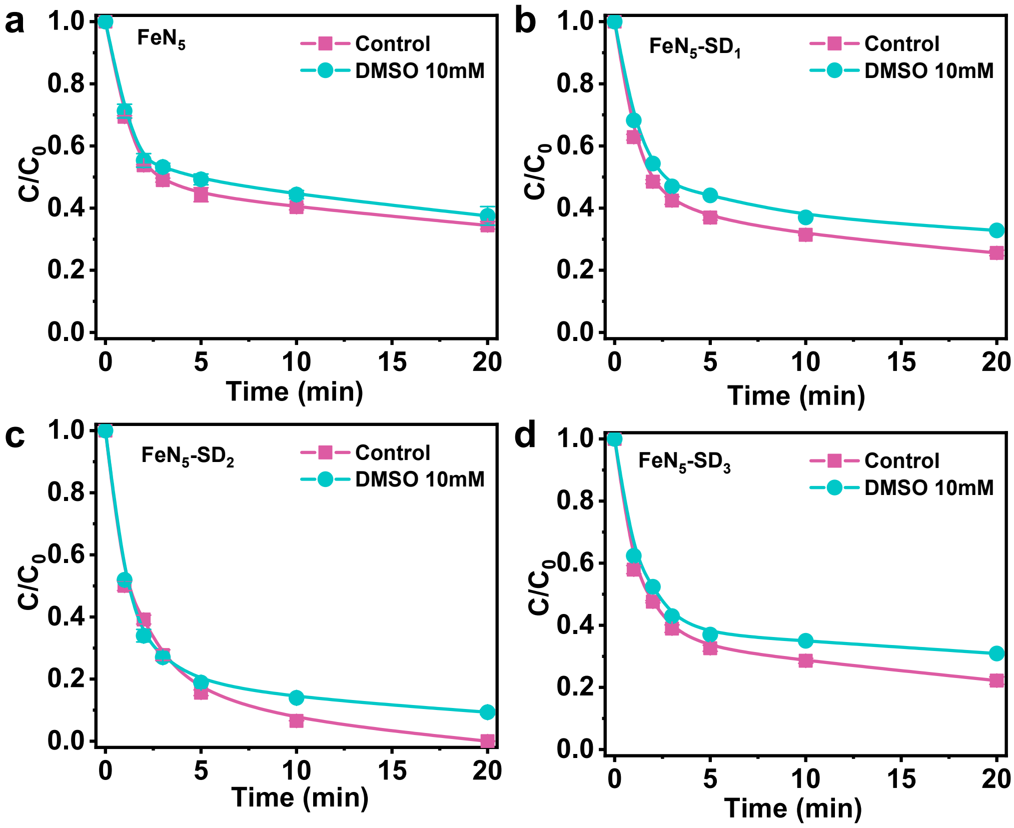

Supplementary Fig. 21 | Investigation of active species. BPA removal in the **a** FeN_5/PMS, **b** FeN_5-SD_1/PMS, **c** FeN_5-SD_2/PMS, and **d** FeN_5-SD_3/PMS system with 10 mM DMSO. Error bars indicate standard deviation derived from three parallel measurements. Routine conditions: [BPA] = 80 μM, [catalyst] = 0.03 g L^{-1}, [PMS] = 0.05 mM, temperature = 25 °C, without pH adjustment. Source data are provided as a Source Data file.

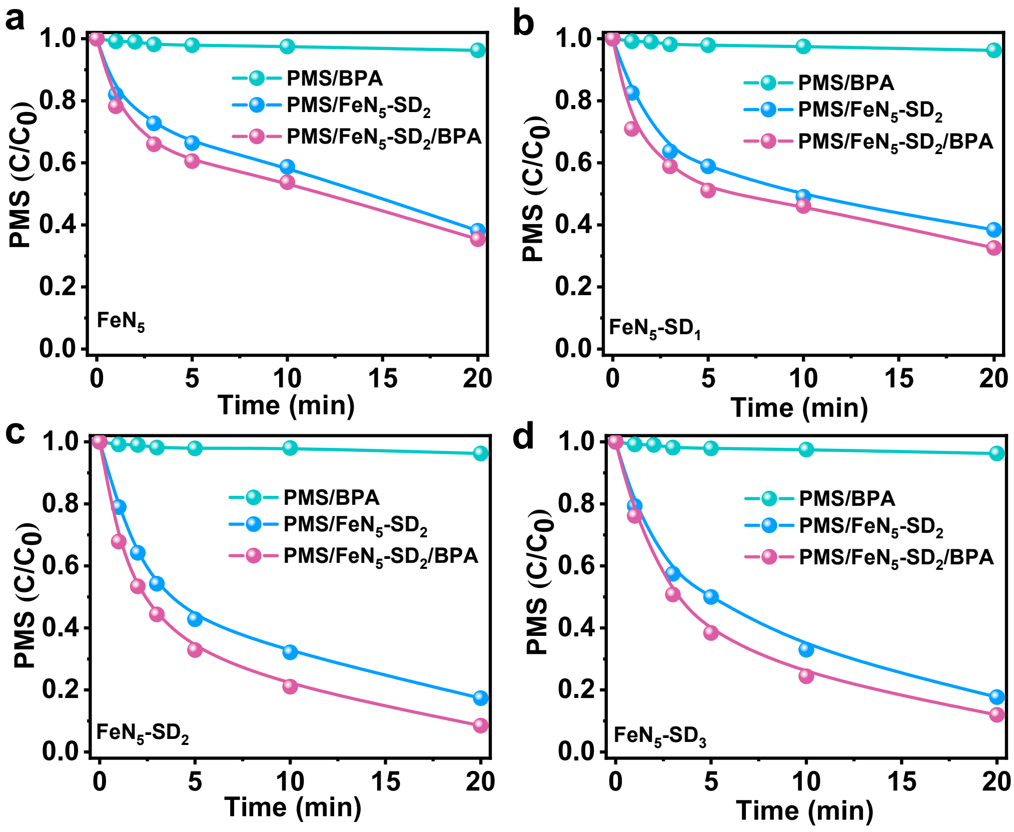

Supplementary Fig. 22 | Identification of the electron transfer pathway. Decomposition rate of PMS in the **a** FeN_5/PMS, **b** FeN_5-SD_1/PMS, **c** FeN_5-SD_2/PMS, and **d** FeN_5-SD_3/PMS system. Routine conditions: [BPA] = 80 μM, [catalyst] = 0.03 g L^{-1}, [PMS] = 0.05 mM, temperature = 25 °C, without pH adjustment. Source data are provided as a Source Data file.

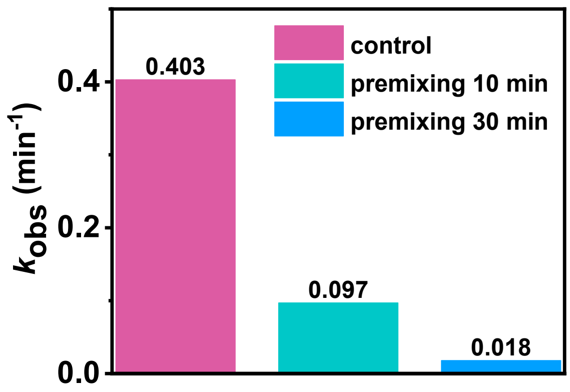

Supplementary Fig. 23 | Identification of the electron transfer pathway. The corresponding k_{obs} in FeN_5-SD_2/PMS system under different premixing time.

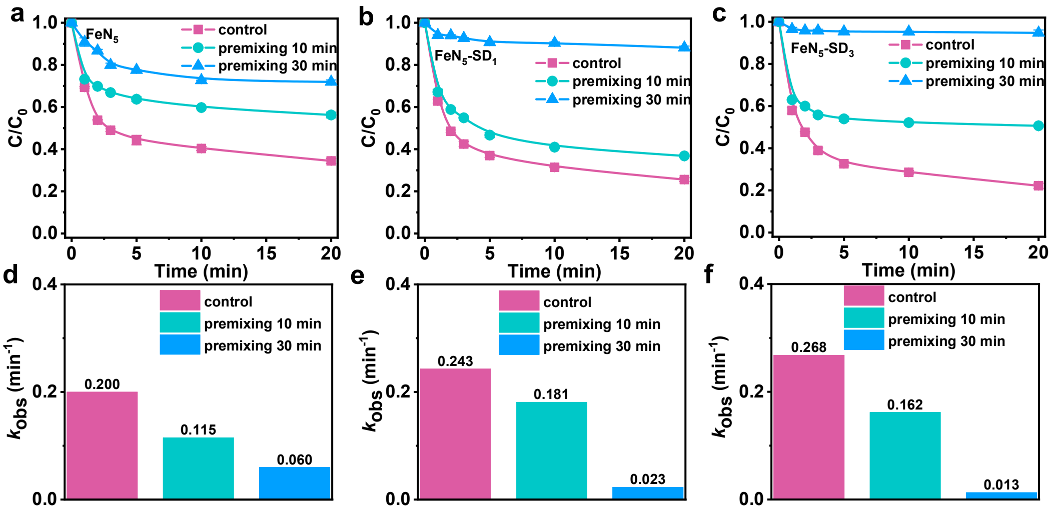

Supplementary Fig. 24 | Identification of the electron transfer pathway. Effect of premixing in the **a** FeN_5/PMS, **b** FeN_5-SD_1/PMS and **c** FeN_5-SD_3/PMS system on BPA removal. Error bars indicate standard deviation derived from three parallel measurements. The corresponding k_{obs} in the **d** FeN_5/PMS, **e** FeN_5-SD_1/PMS and **f** FeN_5-SD_3/PMS system under different premixing time. Routine conditions: [BPA] = 80 μM, [catalyst] = 0.03 g L^{-1}, [PMS] = 0.05 mM, temperature = 25 °C, without pH adjustment. Source data are provided as a Source Data file.

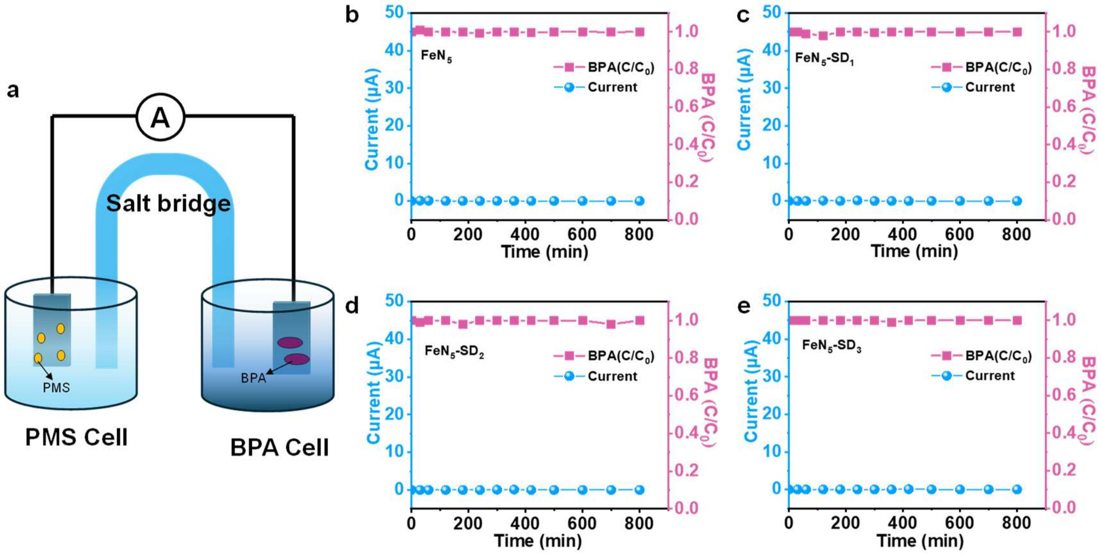

Supplementary Fig. 25 | Identification of the electron transfer pathway. a schematic diagram of the GOP reaction device. The variation of current magnitude and BPA removal during the reaction in the **b** FeN_5/PMS, **c** FeN_5-SD_1/PMS, **d** FeN_5-SD_2/PMS, and **e** FeN_5-SD_3/PMS system. Source data are provided as a Source Data file.

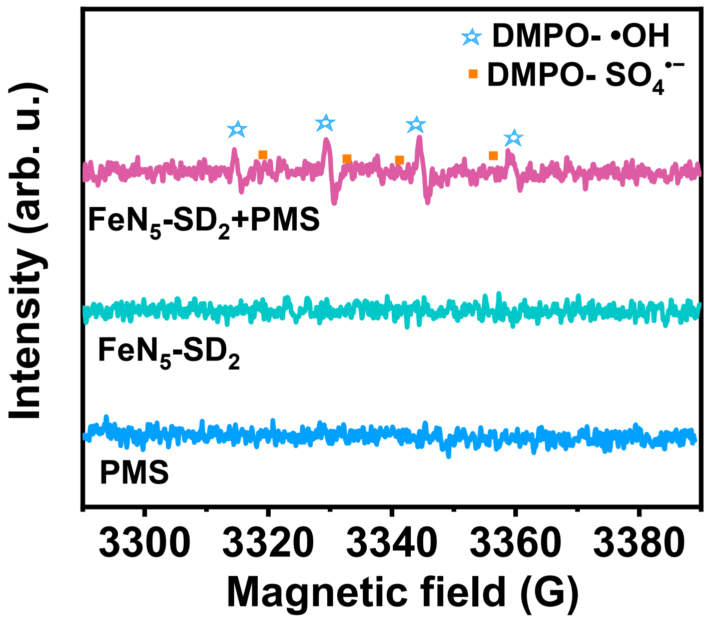

Supplementary Fig. 26 | EPR spectra for detecting $^{•}$OH and $SO_4^{•-}$. EPR spectra of different systems using DMPO in H_2O as the spin-trapping agent for detecting $^{•}$OH and $SO_4^{•-}$. Source data are provided as a Source Data file.

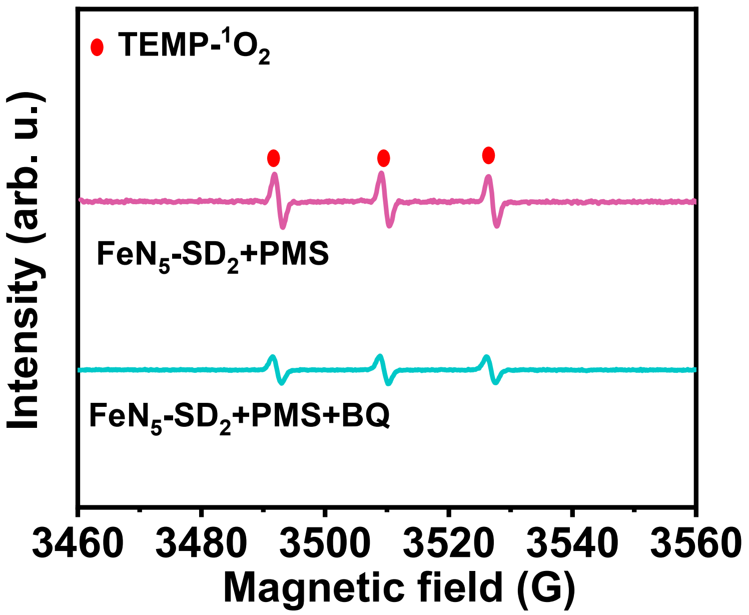

Supplementary Fig. 27 | EPR spectra for detecting $^1O_2$. EPR spectra of $^1O_2$ captured by TEMP in different systems. Source data are provided as a Source Data file.

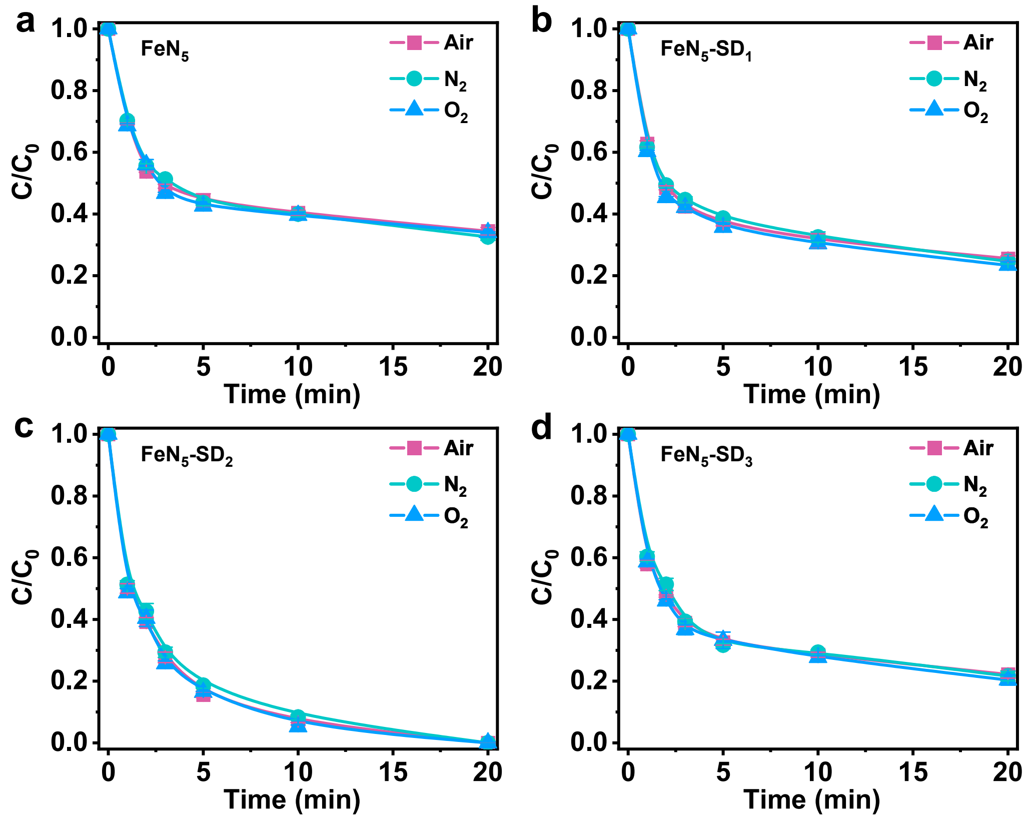

Supplementary Fig. 28 | Investigation of active species. Effect of different gas atmospheres (N_2, air, and O_2) on BPA degradation in **a** FeN_5/PMS, **b** FeN_5-SD_1/PMS, **c** FeN_5-SD_2/PMS, and **d** FeN_5-SD_3/PMS system. Error bars indicate standard deviation derived from three parallel measurements. Routine conditions: [BPA] = 80 μM, [catalyst] = 0.03 g L^{-1}, [PMS] = 0.05 mM, temperature = 25 °C, without pH adjustment. Source data are provided as a Source Data file.

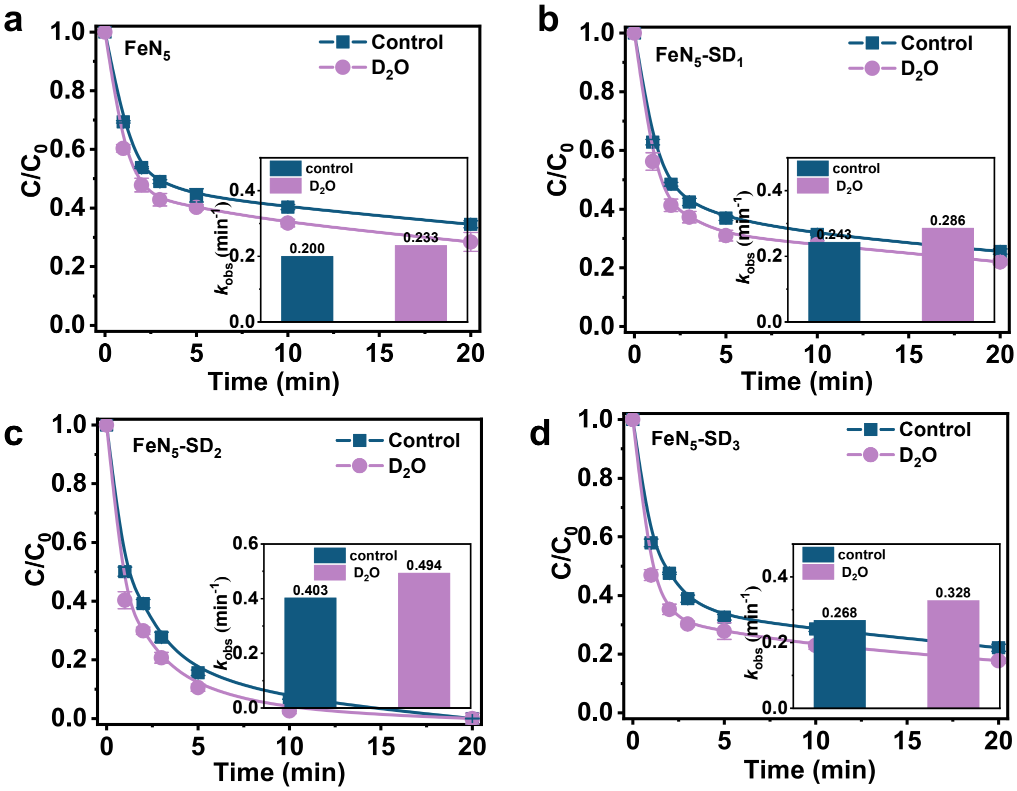

Supplementary Fig. 29 | Investigation of active species. Effect of reaction solvents (H_2O and D_2O) on BPA removal in **a** FeN_5/PMS, **b** FeN_5-SD_1/PMS, **c** FeN_5-SD_2/PMS, and **d** FeN_5-SD_3/PMS system. Error bars indicate standard deviation derived from three parallel measurements. Routine conditions: [BPA] = 80 μM, [catalyst] = 0.03 g L^{-1}, [PMS] = 0.05 mM, temperature = 25 °C, without pH adjustment. Source data are provided as a Source Data file.

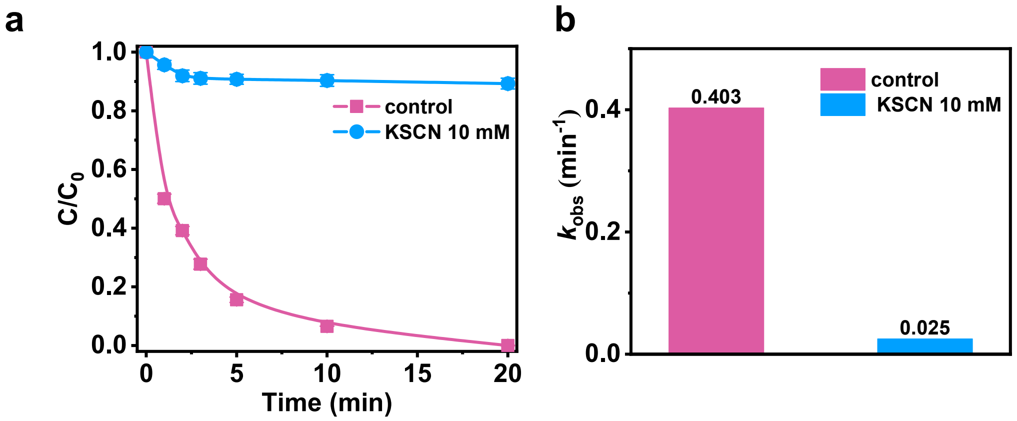

Supplementary Fig. 30 | Masking experiment of catalytic sites. a The effect of KSCN on BPA degradation and **b** the corresponding k_{obs} in FeN_5-SD_2/PMS system. Error bars indicate standard deviation derived from three parallel measurements. Routine conditions: [BPA] = 80 μM, [catalyst] = 0.03 g L^{-1}, [PMS] = 0.05 mM, temperature = 25 °C, without pH adjustment. Source data are provided as a Source Data file.

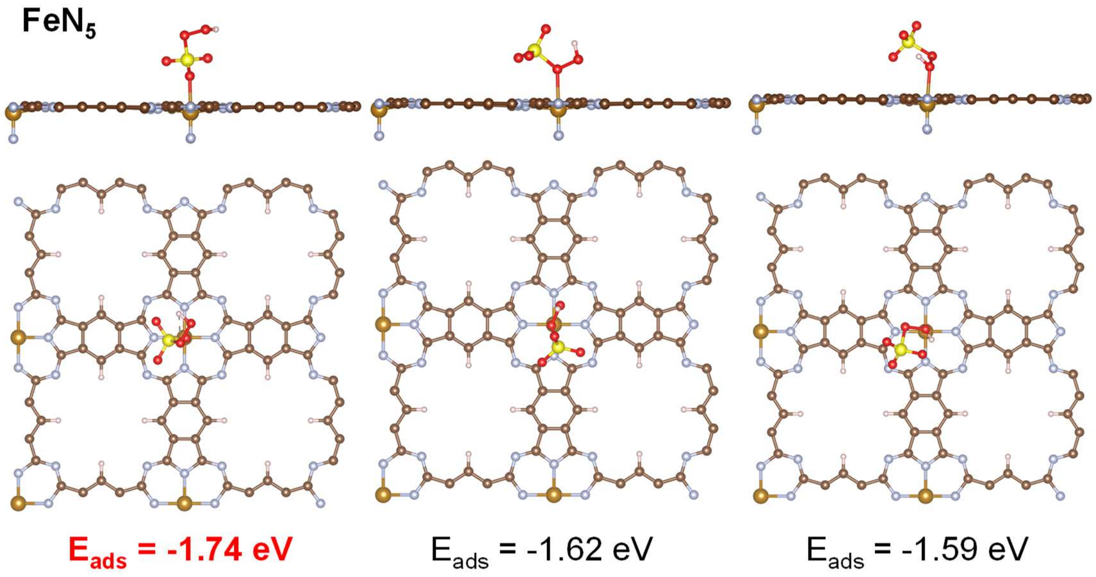

Supplementary Fig. 31 | Investigation of the optimal adsorbed oxygen site. Comparative adsorption energies of PMS oxygen atoms on FeN_5. Crystal structures visualized using VESTA software[1].

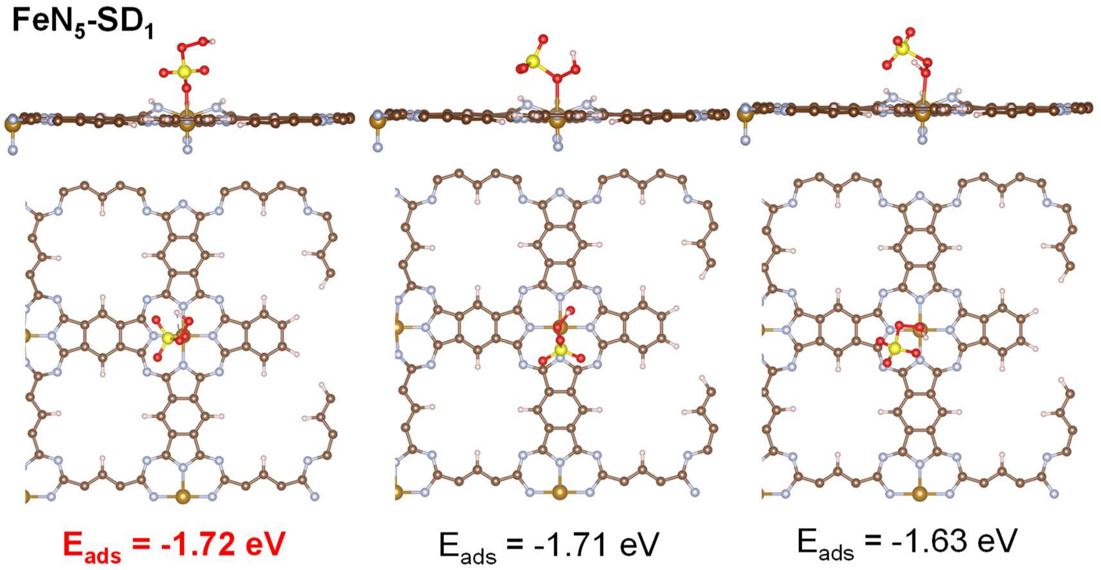

Supplementary Fig. 32 | Investigation of the optimal adsorbed oxygen site. Comparative adsorption energies of PMS oxygen atoms on FeN_5-SD_1. Crystal structures visualized using VESTA software[1].

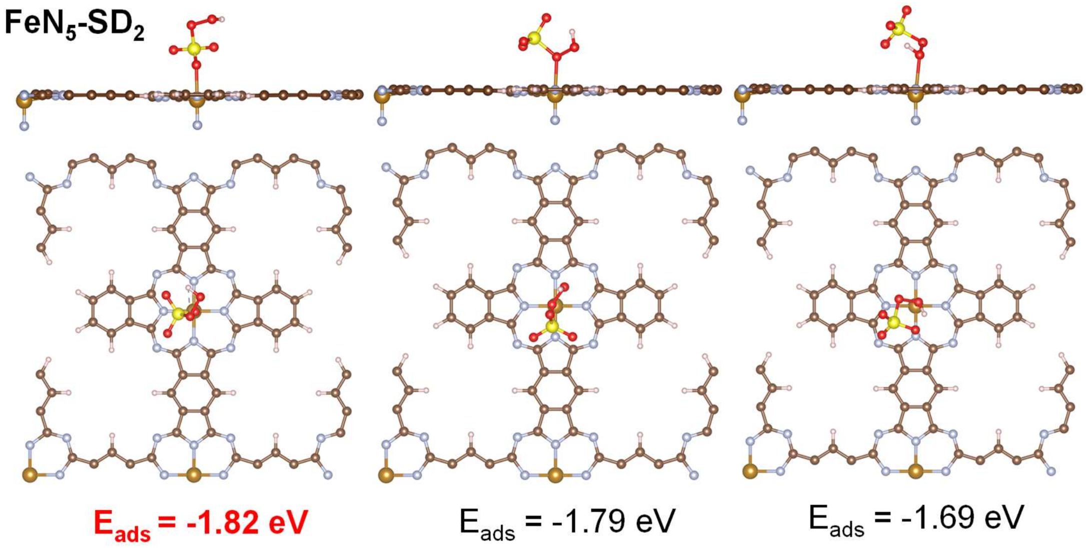

Supplementary Fig. 33 | Investigation of the optimal adsorbed oxygen site. Comparative adsorption energies of PMS oxygen atoms on FeN_5-SD_2. Crystal structures visualized using VESTA software[1].

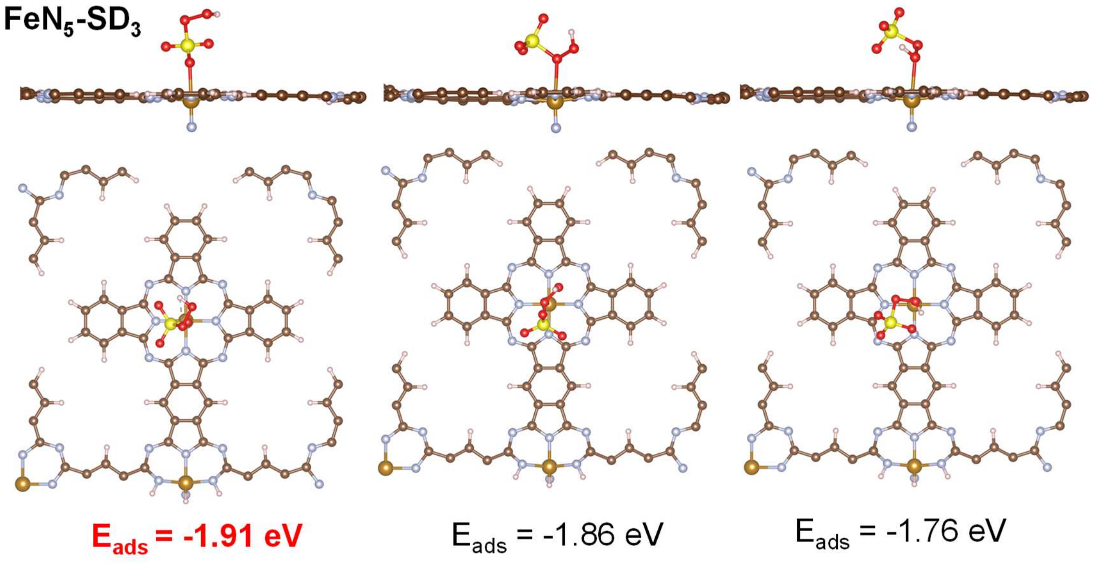

Supplementary Fig. 34 | Investigation of the optimal adsorbed oxygen site. Comparative adsorption energies of PMS oxygen atoms on FeN_5-SD_3. Crystal structures visualized using VESTA software[1].

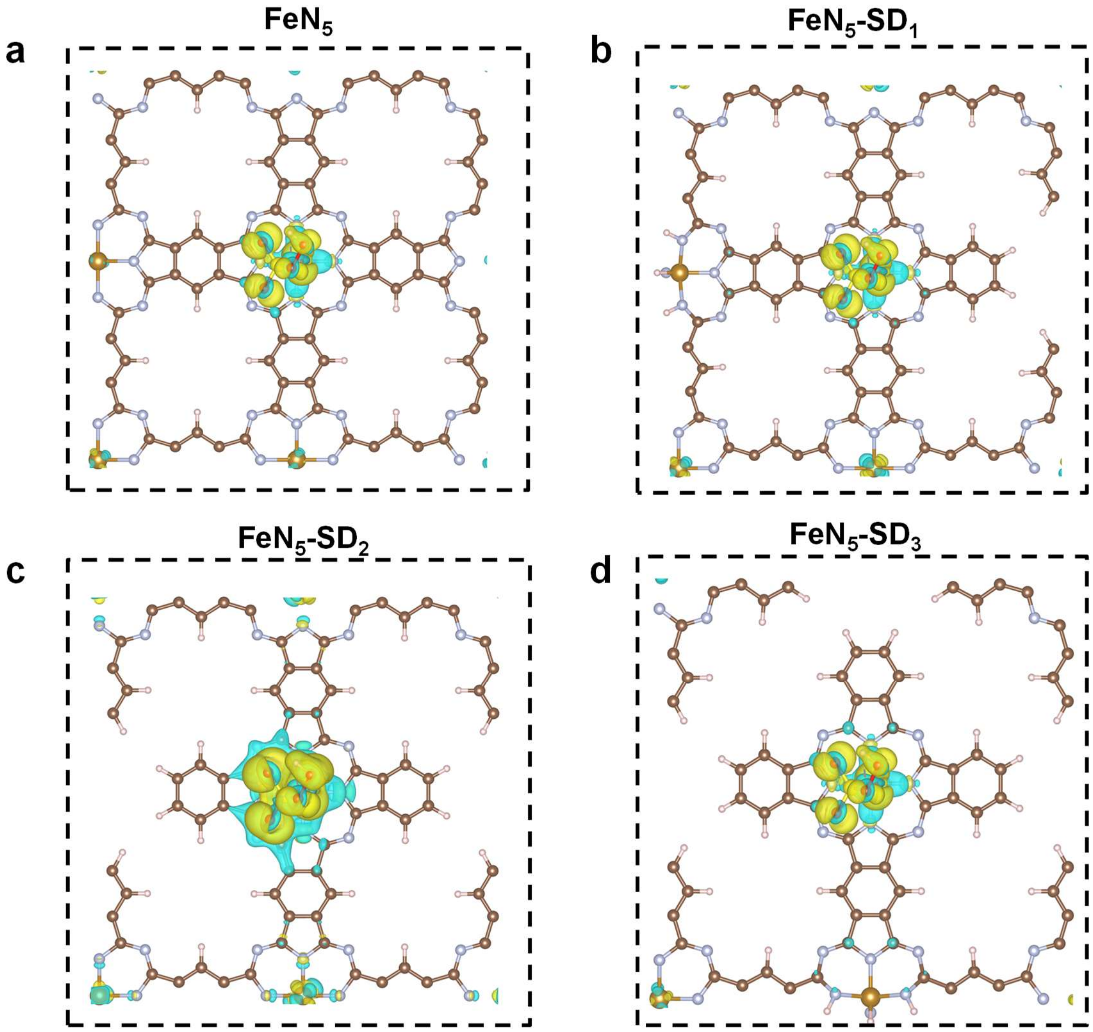

Supplementary Fig. 35 | Charge transfer ability comparison. Top view of the calculated electron density difference diagrams of **a** FeN_5, **b** FeN_5-SD_1, **c** FeN_5-SD_2, and **d** FeN_5-SD_3. Crystal structures visualized using VESTA software[1].

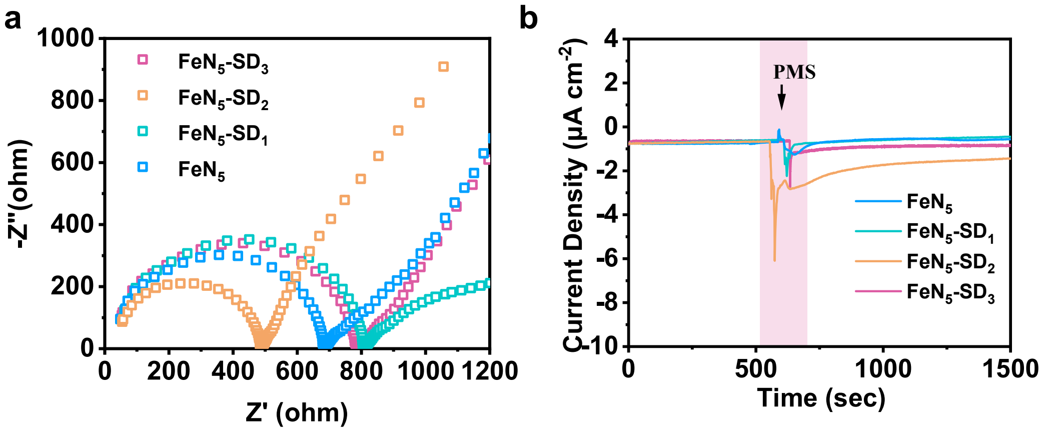

Supplementary Fig. 36 | Electrochemical characterization results. a electrochemical impedance analyses spectra and **b** chronoamperometry analysis of FeN_5, FeN_5-SD_1, FeN_5-SD_2, and FeN_5-SD_3. Source data are provided as a Source Data file.

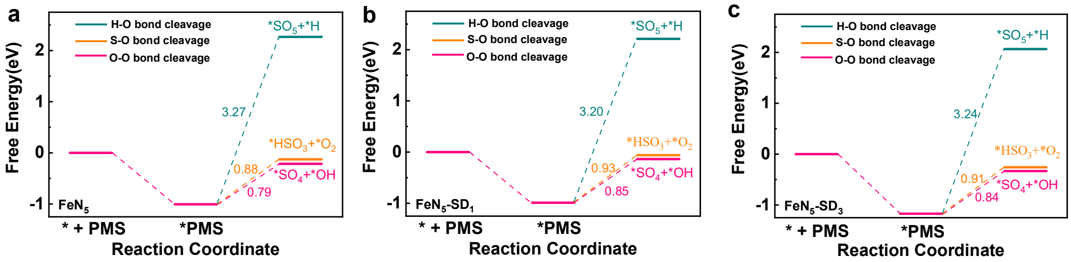

Supplementary Fig. 37 | Comparison of bond dissociation energy barriers. Calculated potential energy diagrams for various bond-cleavage pathways during PMS decomposition over **a** FeN_5, **b** FeN_5-SD_1, and **c** FeN_5-SD_3. Source data are provided as a Source Data file.

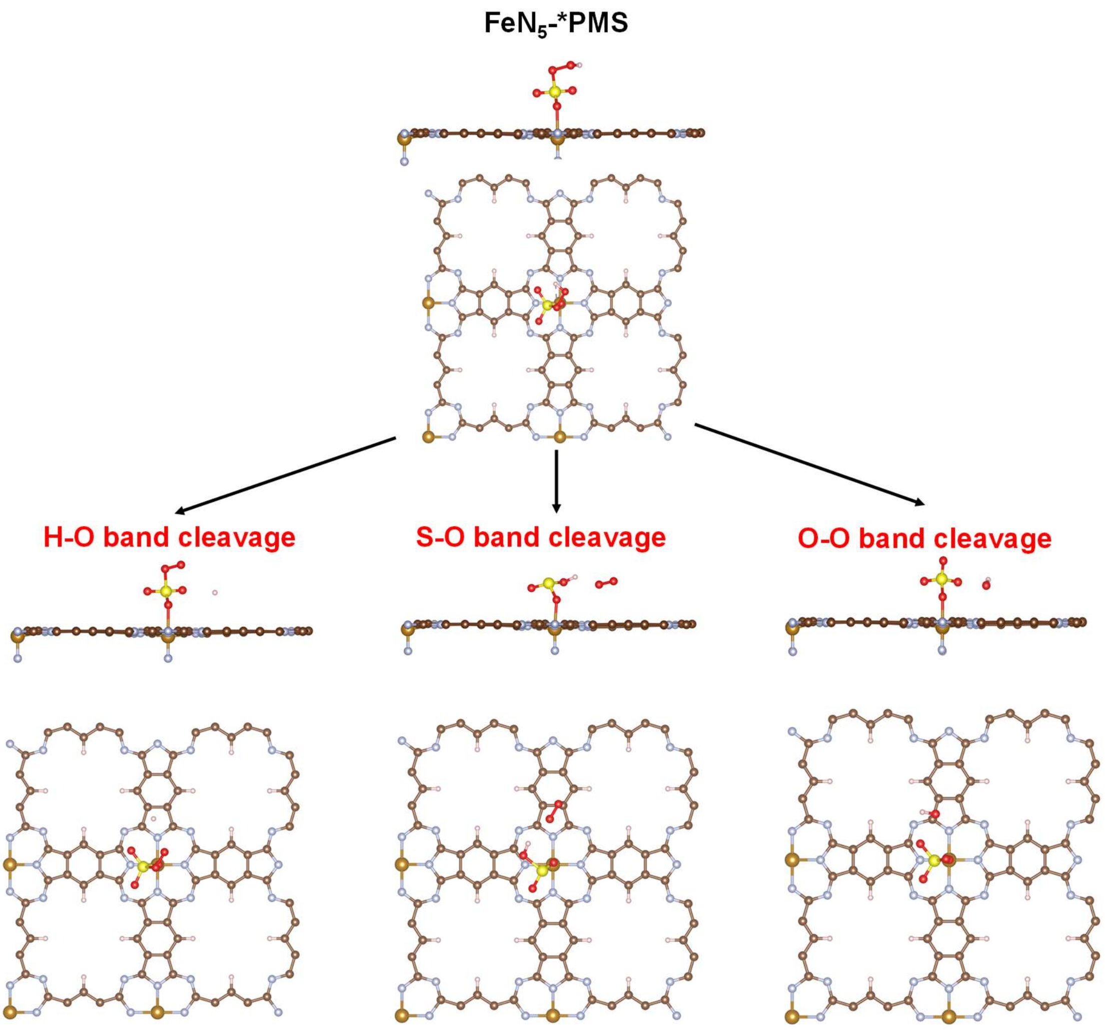

Supplementary Fig. 38 | Comparison of optimal bond cleavage models. Molecular dynamics trajectories of bond-cleavage pathways during PMS decomposition on FeN_5. Crystal structures visualized using VESTA software[1].

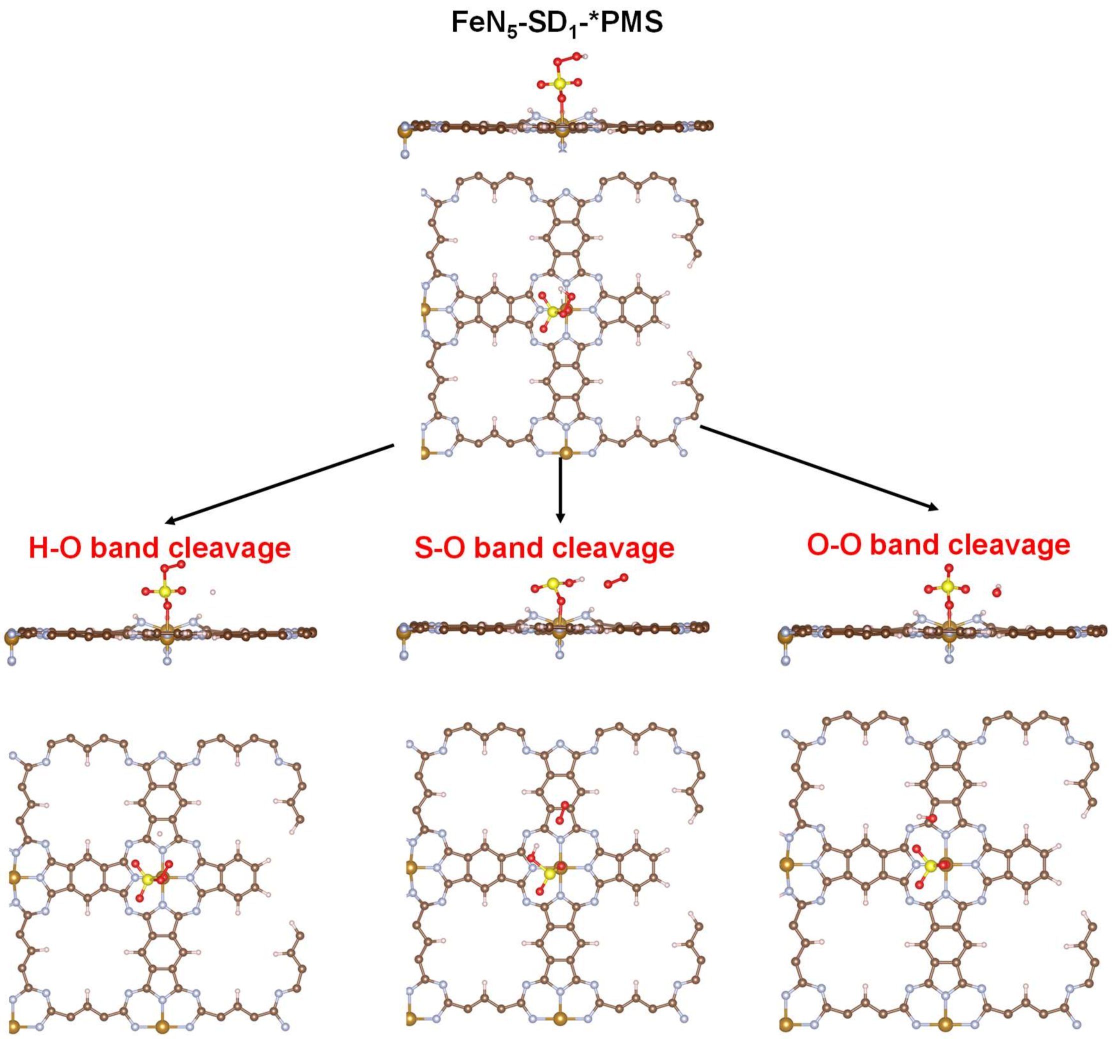

Supplementary Fig. 39 | Comparison of optimal bond cleavage models. Molecular dynamics trajectories of bond-cleavage pathways during PMS decomposition on FeN_5-SD_1. Crystal structures visualized using VESTA software[1].

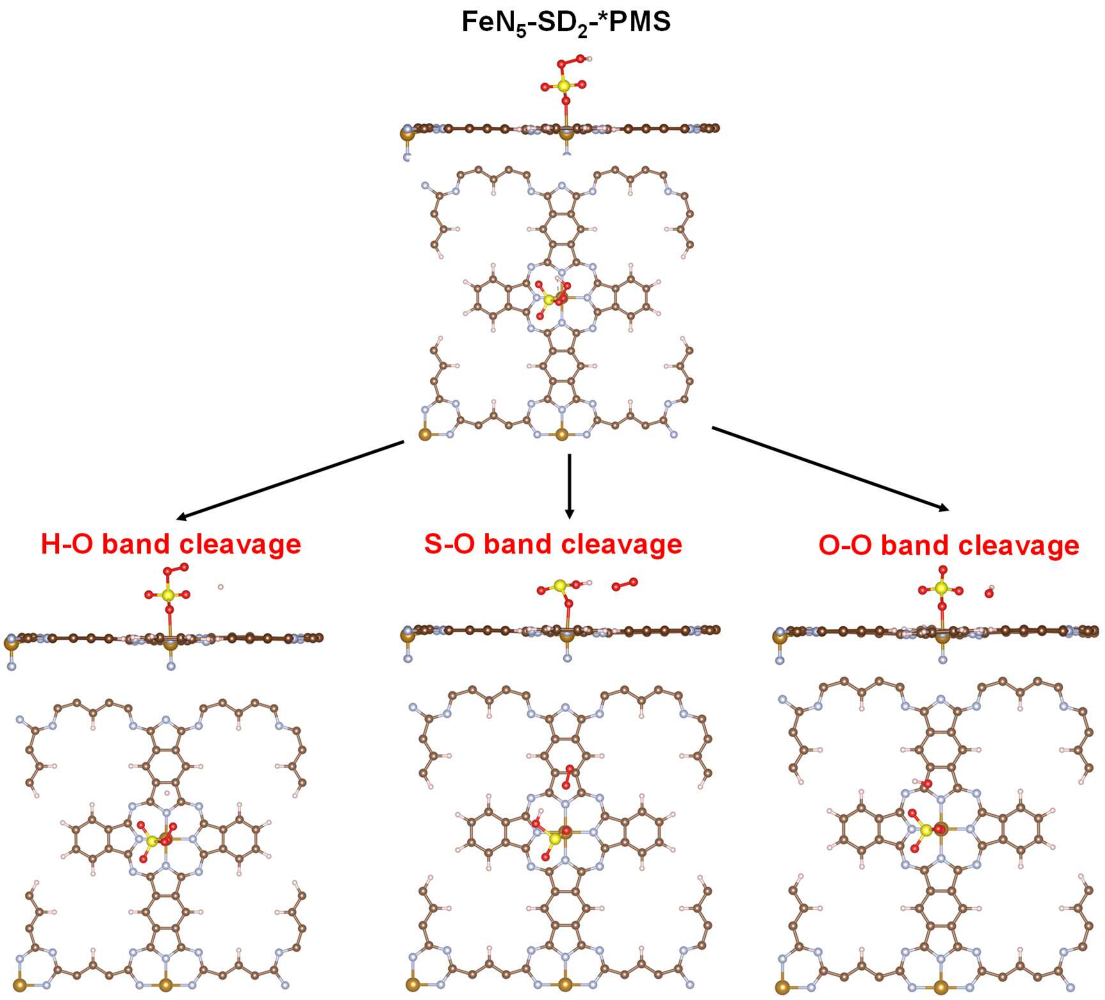

Supplementary Fig. 40 | Comparison of optimal bond cleavage models. Molecular dynamics trajectories of bond-cleavage pathways during PMS decomposition on FeN_5-SD_2. Crystal structures visualized using VESTA software[1].

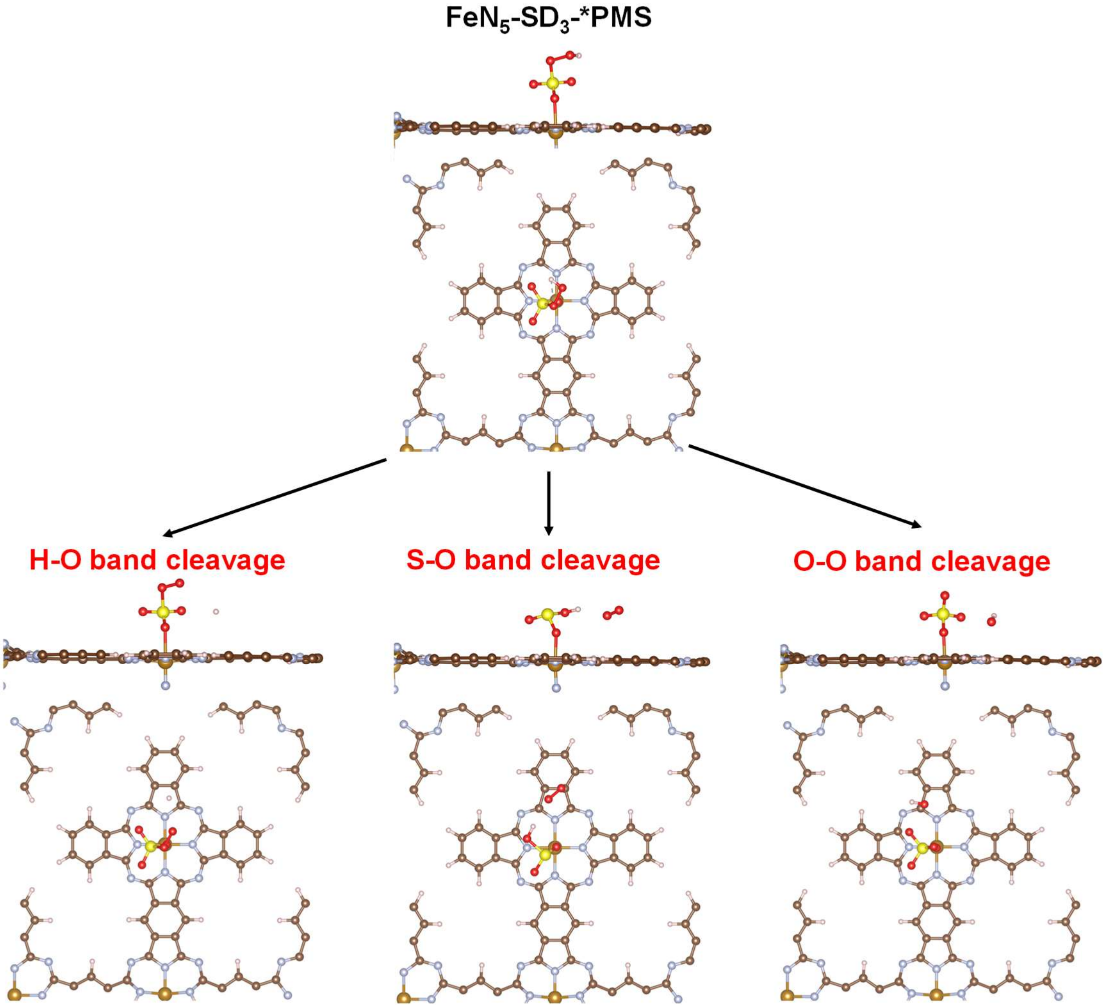

Supplementary Fig. 41 | Comparison of optimal bond cleavage models. Molecular dynamics trajectories of bond-cleavage pathways during PMS decomposition on FeN_5-SD_3. Crystal structures visualized using VESTA software[1].

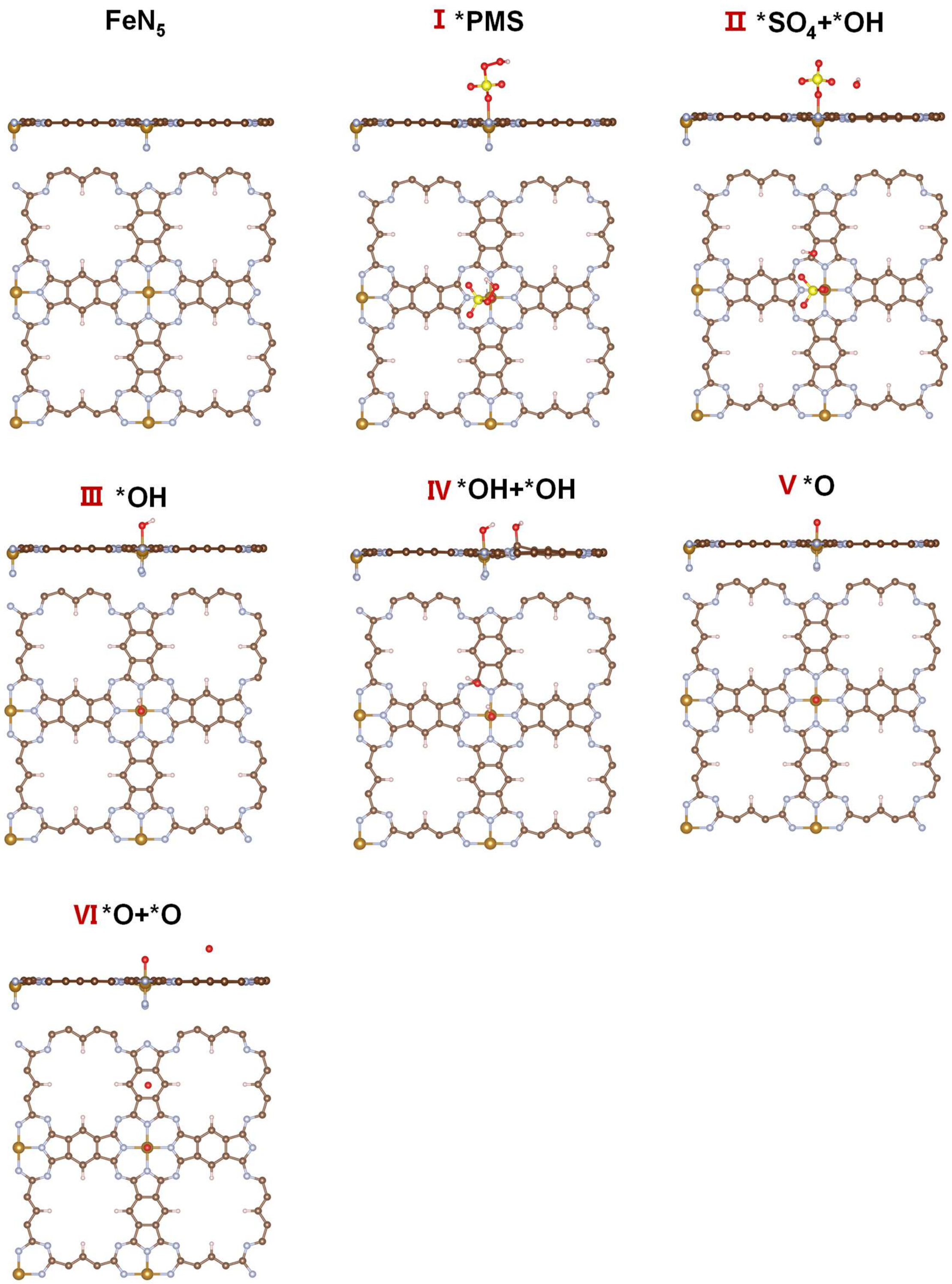

Supplementary Fig. 42 | Kinetic trajectory model. Molecular dynamics trajectories of $^1O_2$ generation during PMS decomposition on FeN_5. Crystal structures visualized using VESTA software[1].

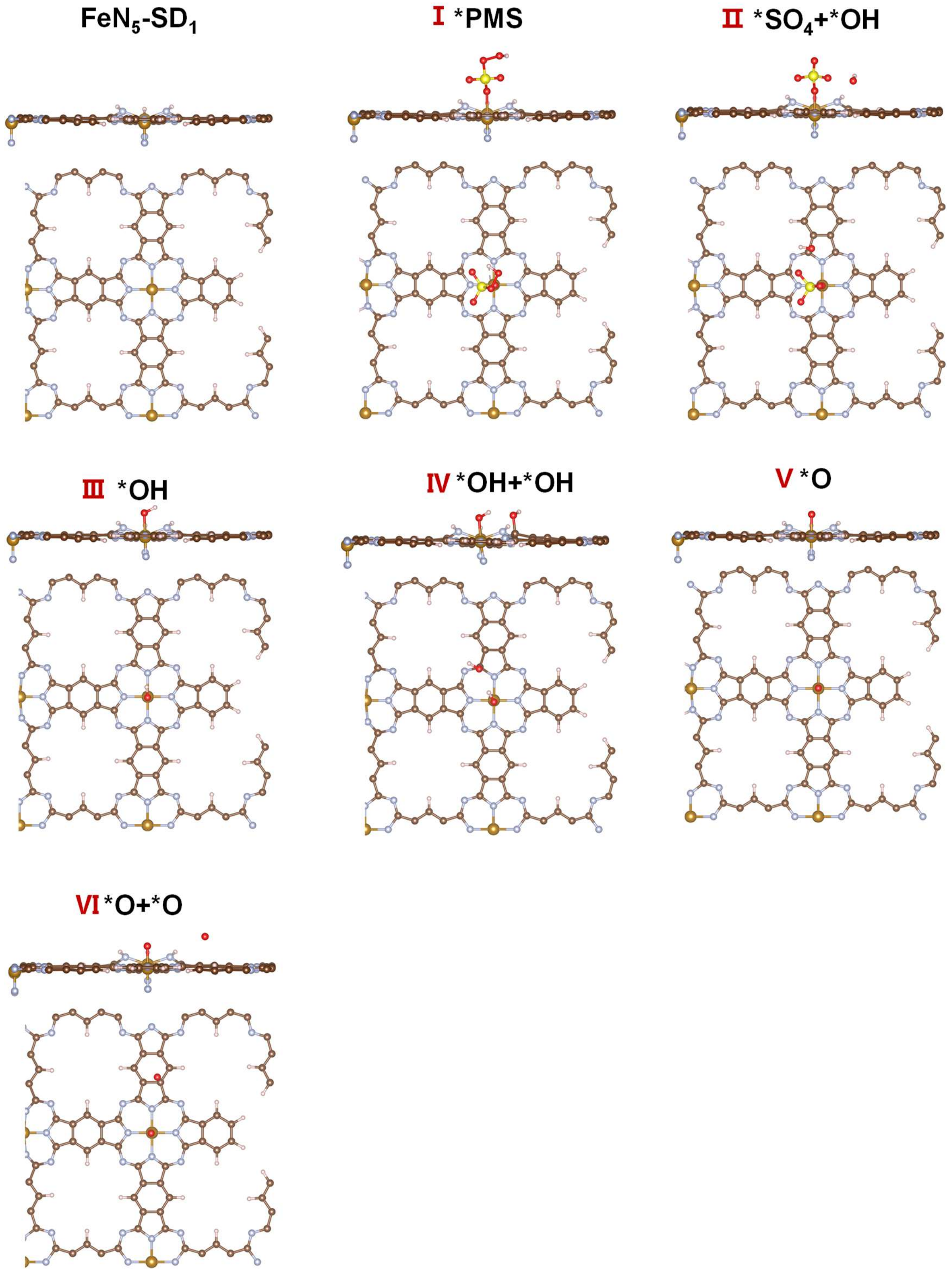

Supplementary Fig. 43 | Kinetic trajectory model. Molecular dynamics trajectories of $^1O_2$ generation during PMS decomposition on FeN_5-SD_1. Crystal structures visualized using VESTA software[1].

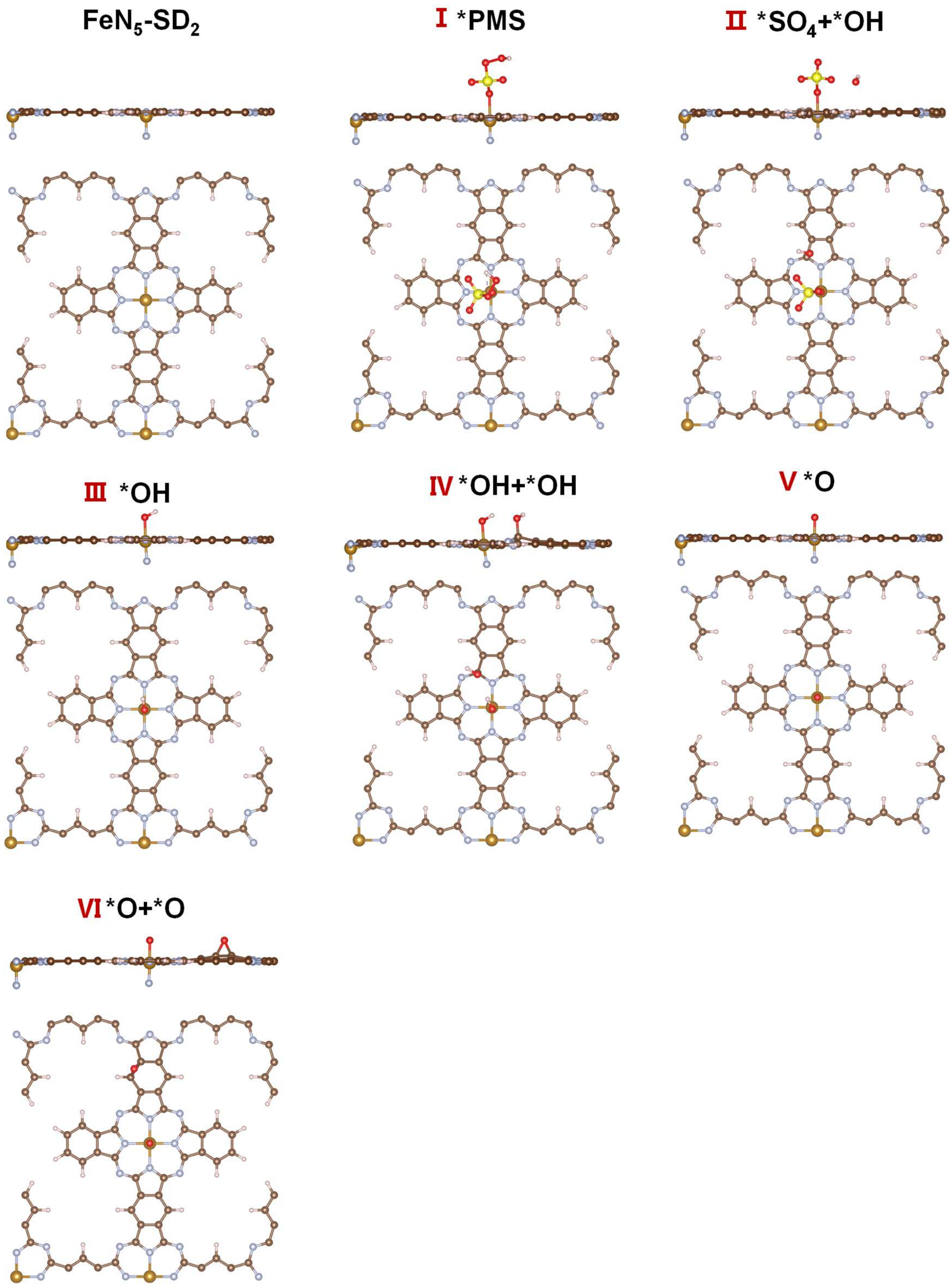

Supplementary Fig. 44 | Kinetic trajectory model. Molecular dynamics trajectories of $^1O_2$ generation during PMS decomposition on FeN_5-SD_2. Crystal structures visualized using VESTA software[1].

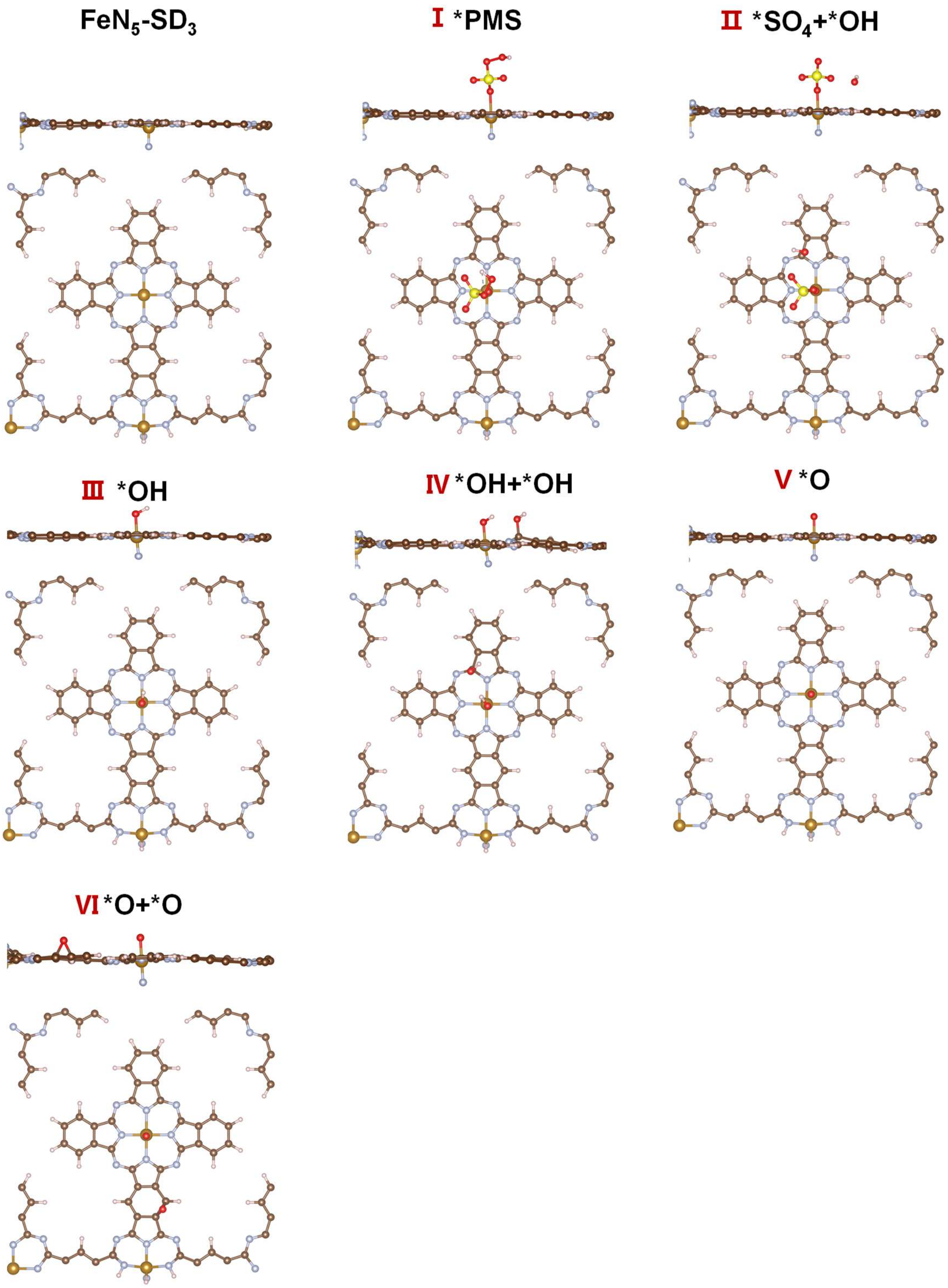

Supplementary Fig. 45 | Kinetic trajectory model. Molecular dynamics trajectories of $^1O_2$ generation during PMS decomposition on FeN_5-SD_3. Crystal structures visualized using VESTA software[1].

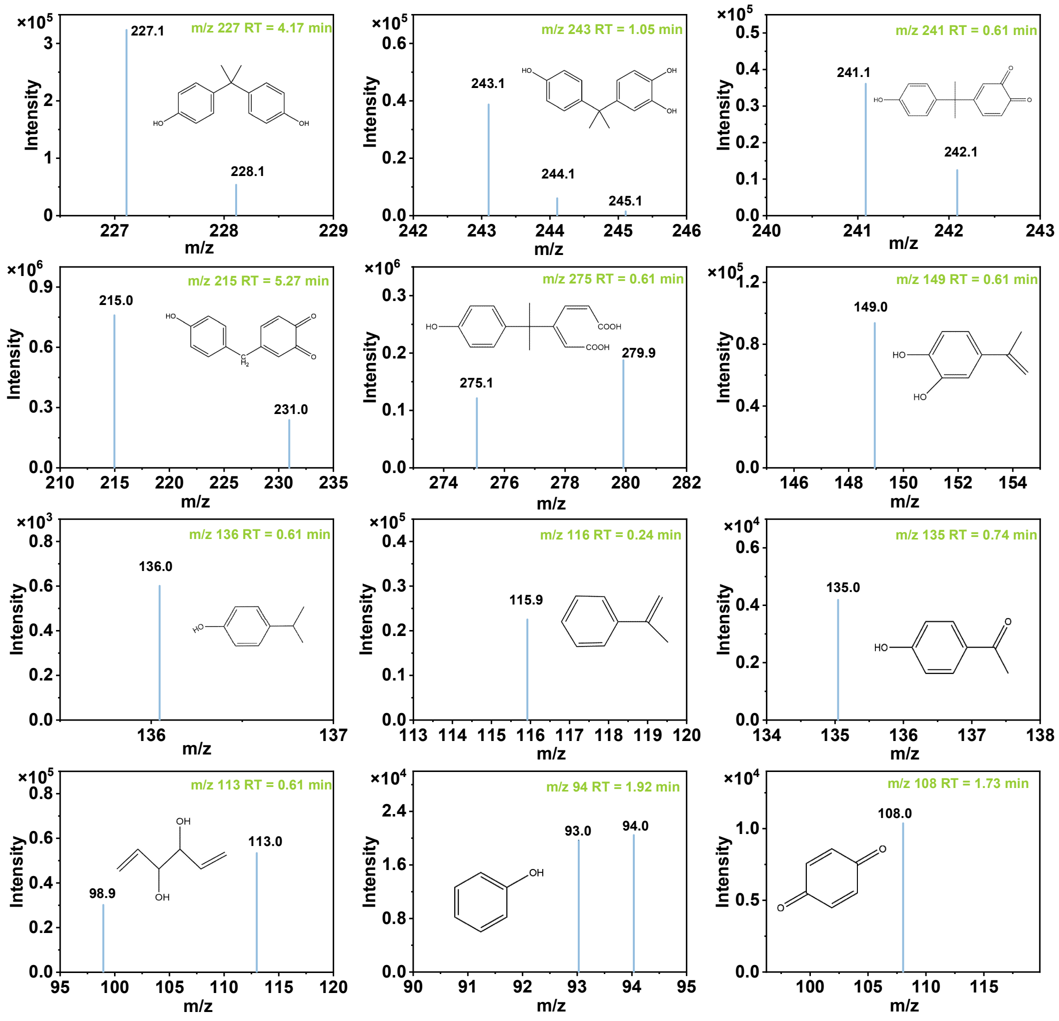

Supplementary Fig. 46 | Structure analysis of intermediates. ESI mass spectra of BPA during degradation in FeN_5-SD_2/PMS system. Source data are provided as a Source Data file.

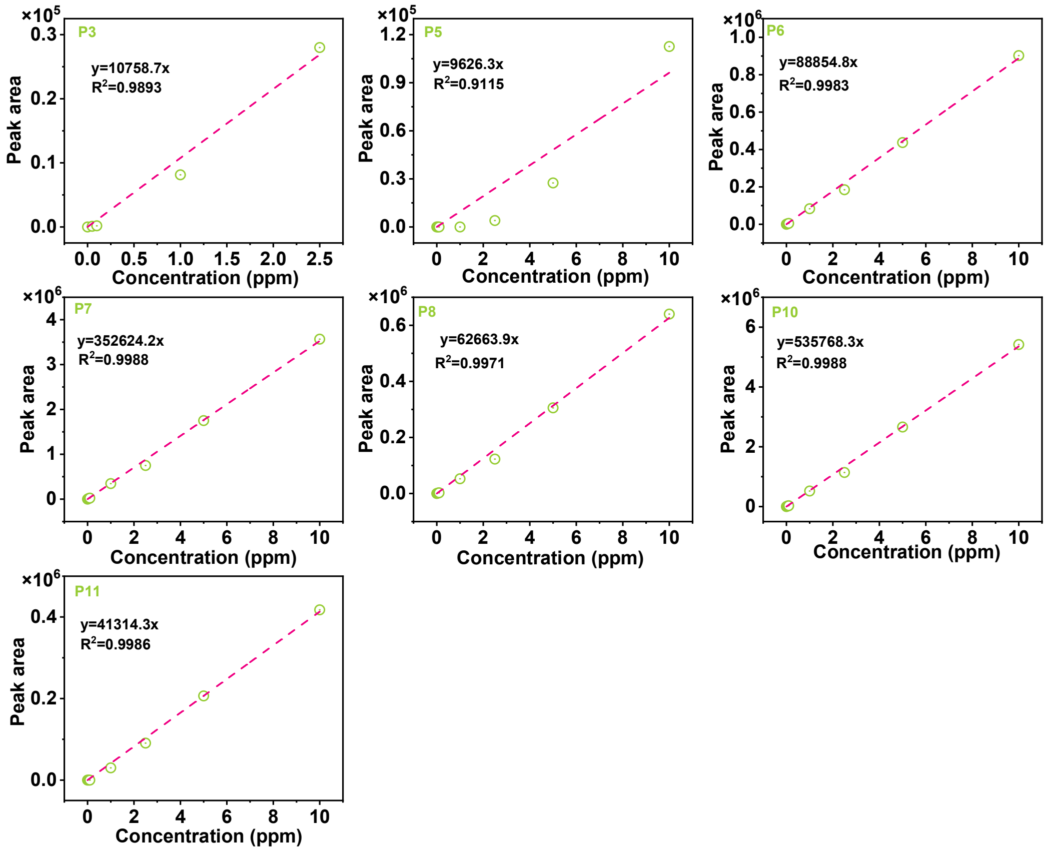

Supplementary Fig. 47 | Quantitative analysis of reaction intermediates. Calibration curves of the intermediates during BPA degradation. Source data are provided as a Source Data file.

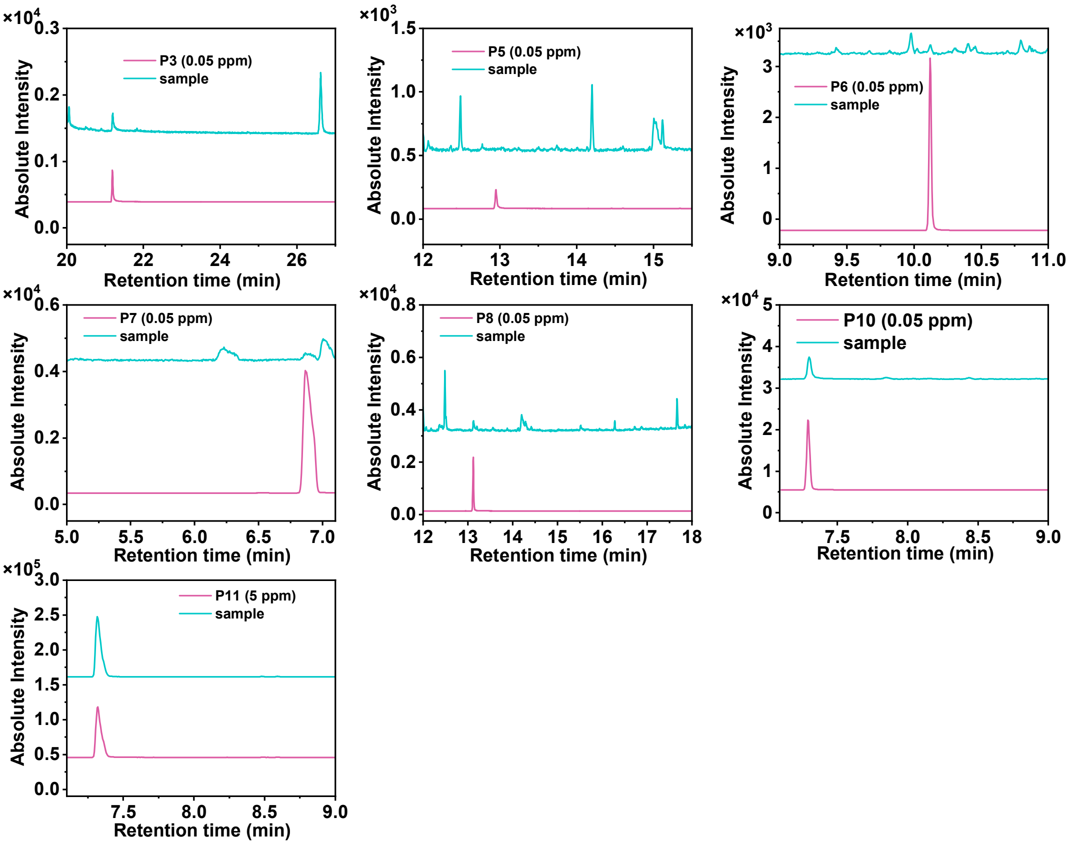

Supplementary Fig. 48 | Quantitative analysis of reaction intermediates. GC/MS chromatograms of the intermediates in the standard solution and the sample from BPA degradation. Source data are provided as a Source Data file.

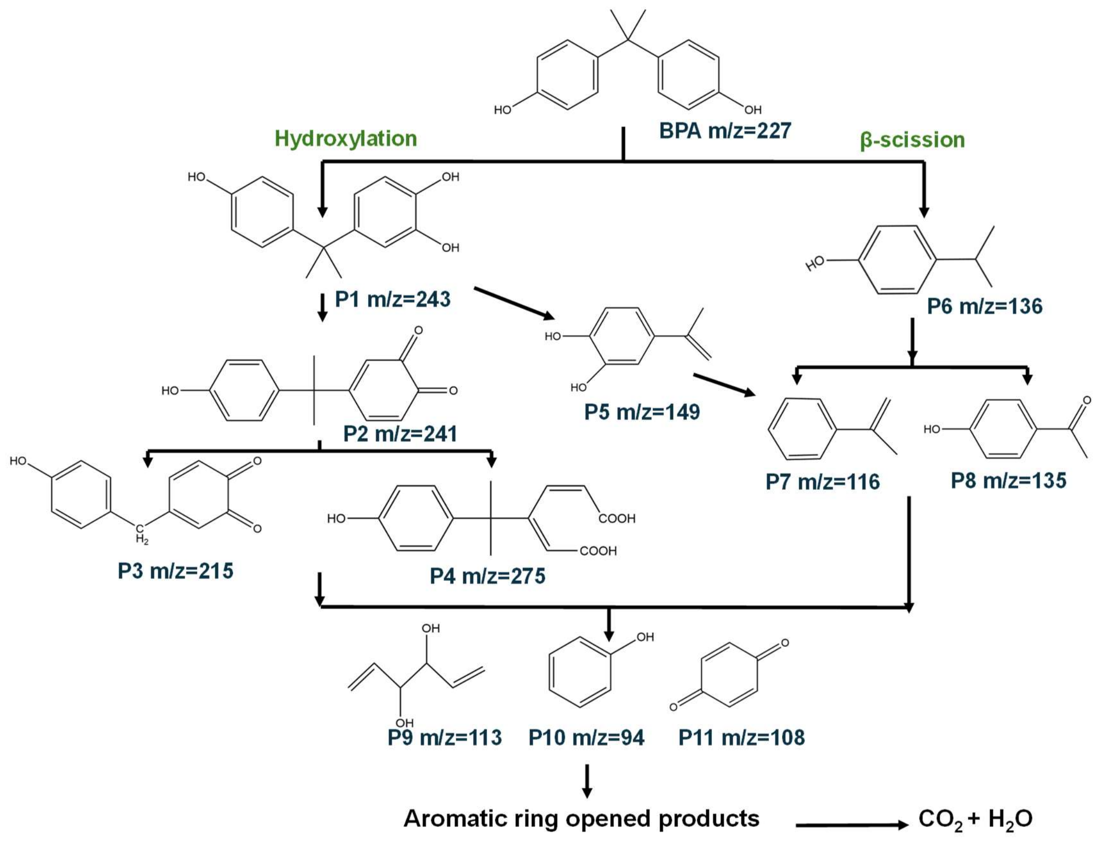

Supplementary Fig. 49 | Analysis of the degradation pathway. Possible degradation pathway diagram of BPA.

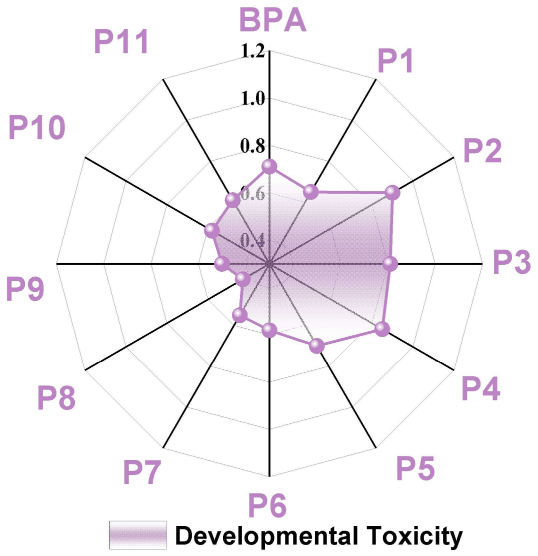

Supplementary Fig. 50 | Toxicity assessment. Developmental toxicity of BPA and its degradation intermediates. Source data are provided as a Source Data file.

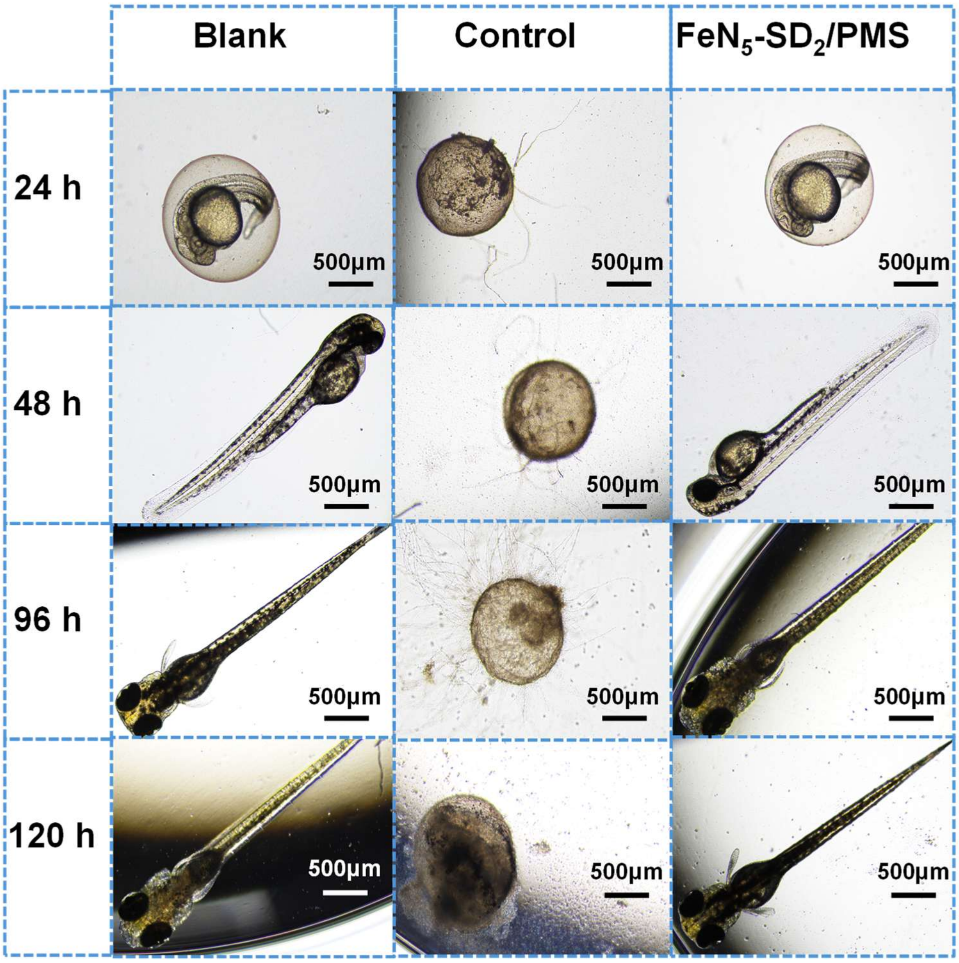

Supplementary Fig. 51 | Comparison of photographs of zebrafish bred in different systems. Images of zebrafish embryos under different treatments: nutrient medium only (Blank), BPA solution (Control), and post-reaction BPA solution from the FeN_5-SD_2/PMS system (FeN_5-SD_2/PMS).

Supplementary Tables

Supplementary Table 1. BET surface area and total pore volume a of FeN_5, FeN_5-SD_1, FeN_5-SD_2 and FeN_5-SD_3

**Catalysts**	**BET specific surface area (m^2 g^{-1})**	**Total pore volume (cm^3 g^{-1})**
FeN_5	43.78	0.1913
FeN_5-SD_1	46.86	0.3856
FeN_5-SD_2	55.63	0.4295
FeN_5-SD_3	65.83	0.4335

Supplementary Table 2. EXAFS fitting parameters at the Fe K-edge for various samples

**Sample**	**Shell**	***CN*** ^{a}	***R*** **(Å)** ^{b}	**σ2 (Å** ^{2} **·10** $^{-3}$ **)** ^{c}	$\mathbf{\Delta E_0}$ **(eV)** ^{d}	**R factor (%)**
Fe Foil	Fe-Fe_1	8*	2.46±0.006	0.0049	4.51±1.12	0.6
	Fe-Fe_2	6*	2.83±0.014	0.0061	6.90±2.12	
Fe_2O_3	Fe-O_1^*	3*	1.93±0.007	0.0069	3.52±0.23	1.1
	Fe-O_2^*	3*	2.07±0.020	0.0040	5.17±1.15	
FePc	Fe-N	4*	1.93±0.021	0.0047	3.64±1.22	0.7
FeN_5-SD_2	Fe-N	4.8±0.2	2.01±0.018	0.0018	3.72±1.09	1.5

^{a}*CN*, coordination number; ^{b}*R*, distance between absorber and backscatter atoms; $^{c}\sigma^2$, Debye-Waller factor to account for both thermal and structural disorders; $^{d}\Delta E_0$, inner potential correction; *R* factor indicates the goodness of the fit. $S0^2$ was fixed to 0.78, according to the experimental EXAFS fit of Fe foil by fixing CN as the known crystallographic value.

Supplementary Table 3. Comparison of molar iron-normalized rate constants ($K_{per\text{-}mol\ Fe}$) for FeN_5-Based PMS systems.

**Catalysts**	**$m_{cat.}$ ($g{\cdot}L^{-1}$)**	**ω_{metal} (%)**	**k_{obs} (min^{-1})**	**$K_{per\text{-}mol\ Fe}$ ($\times 10^4\ min^{-1}{\cdot}M^{-1}$)**
FeN_5	0.03	2.88	0.200	1.30
FeN_5-SD_1	0.03	2.59	0.243	1.75
**FeN_5-SD_2**	**0.03**	**2.21**	**0.403**	**3.40**
FeN_5-SD_3	0.03	1.69	0.268	2.96

Supplementary Table 4. Comparison of area-normalized rate constants ($K_{per\text{-}area}$) for FeN_5-based PMS systems with varying defect densities.

**Catalysts**	**BET (m^2/g)**	**k_{obs} (min^{-1})**	**$K_{per\text{-}area}$ ($min^{-1}/(m^2 \cdot g^{-1})$)**
FeN_5	43.7834	0.200	0.0046
FeN_5-SD_1	46.8609	0.243	0.0052
**FeN_5-SD_2**	**55.6287**	**0.403**	**0.0072**
FeN_5-SD_3	65.8330	0.268	0.0041

Supplementary Table 5. Catalytic performances of SAC-based PMS systems regulated *via* coordination environment engineering.

**Catalysts (g L^{-1})**	**Δn(pollutants) ($\times 10^{-6}$ mol)**	**ω_{metal} (%)**	**Reaction time (min)**	**TOF (min^{-1})**	**Ref.**
$Fe-N_3C_1$ (0.06)	SIZ (19.6)	0.75	30	0.081	2
$Fe-N_2C_2$ (0.06)	SIZ (17)	0.84	30	0.063	2
Co-OCN (0.03)	APAP (13)	7.25	60	0.006	3
Fe–N–C (0.10)	BPA (100)	2.00	20	0.140	4
Co-N_5/CNT (0.03)	SMZ (40)	1.39	30	0.189	5
$CNFe_2$-0.6 (0.08)	SMZ (79)	16.64	8	0.042	6
Fe_1/CN (0.05)	4-CP (100)	11.2	10	0.100	7
Fe_{SA}-N/C-20 (0.15)	BPA (88)	0.88	20	0.187	8
FeSA-N/O-C (0.10)	BPA (66)	1.28	45	0.064	9
Co-N_3 (0.10)	CBZ (42)	2.51	6	0.165	10
CoN_3O_1 (0.10)	CIP (15)	0.51	20	0.087	11
Fe-N_4-C (0.10)	NPX (43)	1.50	10	0.161	12
SA-FeN_5 (0.20)	APAP (30)	2.63	12	0.027	13
FeN_5 (0.03)	BPA (52)	2.88	20	0.169	This work
FeN_5-SD_1 (0.03)	BPA (56)	2.59	20	0.202	This work
FeN_5-SD_2 **(0.03)**	**BPA (80)**	**2.21**	**20**	**0.338**	**This work**
FeN_5-SD_3 (0.03)	BPA (59)	1.69	20	0.325	This work

Supplementary Table 6. Quantitative analysis of degradation intermediates by GC-MS.

Product	Chemical name	Selected Quantitative ions (m/z)	Retention time (min)	Peak area	Concentration (ng/mL)
P3	4-(4-hydroxybenzyl)cyclohexa-3,5-diene-1,2-dione	214	21.20	806	3.75
P5	4-(prop-1-en-2-yl)benzene-1,2-diol	150	12.95	ND	ND
P6	4-isopropylphenol	136	10.12	238	0.14
P7	prop-1-en-2-ylbenzene	116	6.87	396	0.06
P8	1-(4-hydroxyphenyl)ethan-1-one	136	13.12	740	0.59
P10	Phenol	94	7.30	11899	1.11
P11	benzoquinone	108	7.32	242230	293.13

Supplementary Table 7. Acute and chronic toxicity results of BPA and its degradation products predicted using the Ecological Structure-Activity Relationships system.

**Degradation intermediates**	**Acute toxicity (mg/L)**			**Chronic toxicity (mg/L)**		
	**Fish**	**Daphnid**	**Green Algae**	**Fish**	**Daphnid**	**Green Algae**
	**LC_{50}**	**LC_{50}**	**EC_{50}**	**Chv**	**Chv**	**Chv**
BPA	6.27	4.15	5.78	0.733	0.617	2.12
P1	18.1	11.5	13.3	2.01	1.51	4.42
P2	13.3	8.50	10.4	1.50	1.16	3.58
P3	71.1	42.0	37.0	7.29	4.59	10.6
P4	906	536	473	93.0	58.6	136
P5	45.5	27.0	24.2	4.69	2.98	7.00
P6	15.1	9.37	10.1	1.64	1.17	3.23
P7	4.92	3.19	4.12	0.563	0.451	1.45
P8	593	313	171	53.1	24.8	38.0
P9	2870	1400	555	235	89.3	103
P10	212	115	71.1	19.6	9.89	16.8
P11	3330	1610	614	269	100	112

Toxicity categories based on GHS: Very toxic (≤1 mg/L), Toxic (1–10 mg/L), Harmful (10–100 mg/L), Not harmful (>100 mg/L).

Supplementary References

1. Momma, K. & Izumi, F. VESTA 3 for three-dimensional visualization of crystal, volumetric and morphology data, *J. Appl. Crystallogr.* **44**, 1272–1276 (2011).

2. Wu, Z. et al. Facilely tuning the first-shell coordination microenvironment in iron single-atom for Fenton-like chemistry toward highly efficient wastewater purification. *Environ. Sci. Technol.* **57**, 14046-14057 (2023).

3. Wu, Q. Y., Yang, Z. W., Wang, Z. W. & Wang, W. L. Oxygen doping of cobalt-single-atom coordination enhances peroxymonosulfate activation and high-valent cobalt–oxo species formation. *Pro. Natl. Acad. Sci.* **120**, e2219923120 (2023).

4. Cheng, C. et al. Generation of FeIV=O and its contribution to Fenton-like reactions on a single-atom iron−N−C catalyst. *Angew. Chem.* **135**, e202218510 (2023).

5. Xie, M. et al. Single-atom $Co-N_5$ catalytic sites on carbon nanotubes as peroxymonosulfate activator for sulfamerazine degradation via enhanced electron transfer pathway. *Sep. Purif. Technol.* **304**, 122398 (2023).

6. Zhu, C. et al. Two-step pyrolysis to anchor ultrahigh-density single-atom FeN_5 sites on carbon nitride for efficient Fenton-like catalysis near 0°C. *Appl. Catal. B* **319**, 121900 (2022).

7. Zhang, L. S. et al. Carbon nitride supported high-loading fe single-atom catalyst for activation of peroxymonosulfate to generate $^1O_2$ with 100 % selectivity.

Angew. Chem. Int. Ed. **60**, 21751-21755 (2021).

8. Yang, T., Fan, S., Li, Y. & Zhou, Q. Fe-N/C single-atom catalysts with high density of Fe-N_x sites toward peroxymonosulfate activation for high-efficient oxidation of bisphenol A: Electron-transfer mechanism. *Chem. Eng. J* **419**, 129590 (2021).

9. Chen, T. et al. Boosting peroxymonosulfate activation by porous single-atom catalysts with FeN_4O_1 configuration for efficient organic pollutants degradation. *Chem. Eng. J* **450**, 138469 (2022).

10. Yin, K. et al. Microenvironment modulation of cobalt single-atom catalysts for boosting both radical oxidation and electron-transfer process in Fenton-like system. *Appl. Catal. B* **329**, 122558 (2023).

11. Wang, Z. et al. Cobalt single atoms anchored on oxygen-doped tubular carbon nitride for efficient peroxymonosulfate activation: simultaneous coordination structure and morphology modulation. *Angew. Chem.* **134**, e202202338 (2022).

12. Yin, K. et al. High-loading of well dispersed single-atom catalysts derived from Fe-rich marine algae for boosting Fenton-like reaction: Role identification of iron center and catalytic mechanisms. *Appl. Catal. B* **336**, 122951 (2023).

13. Liu, C. et al. The “4 + 1” strategy fabrication of iron single-atom catalysts with selective high-valent iron-oxo species generation. *Pro. Natl. Acad. Sci.* **121**, e2322283121 (2024).
