# Supplementary material for: Remote tuning of single-atom Fe-N5 sites via high-coordination defects for enhanced Fenton-like water decontamination
Source: Nat Commun. 2025 Nov 25;16:10455. doi: 10.1038/s41467-025-65425-4 (PMC12647131; doi:10.1038/s41467-025-65425-4)
Supplement: Supplementary file 2 — Description of Additional Supplementary Files [file 41467_2025_65425_MOESM2_ESM.pdf]

Description of Additional Supplementary Files

File Name: Supplementary Data 1

Description: Supplementary Data 1 provides the atomic coordinates of the catalyst's electronic structure as required, with figure legends including 'Serial number,' 'Element,' 'Element sequence number,' 'X-axis spatial position,' 'Y-axis spatial position,' and 'Z-axis spatial position.'
