# Supplementary material for: Remote tuning of single-atom Fe-N5 sites via high-coordination defects for enhanced Fenton-like water decontamination
Source: Nat Commun. 2025 Nov 25;16:10455. doi: 10.1038/s41467-025-65425-4 (PMC12647131; doi:10.1038/s41467-025-65425-4)
Supplement: Supplementary file 3 — Supplementary Data 1 [file 41467_2025_65425_MOESM3_ESM.docx]

**DFT-Optimized FeN_5_ Atomic Coordinates**

| **Serial number** | **element** | **Element sequence number** | **x-axis space position** | **y-axis space position** | **z-axis space position** |
| --- | --- | --- | --- | --- | --- |
| 1 | N | N1 | 0.02094 | 0.61171 | 0.20222 |
| 2 | N | N2 | 0.93011 | 0.52082 | 0.20347 |
| 3 | N | N3 | 0.11171 | 0.52085 | 0.20333 |
| 4 | N | N4 | 0.02089 | 0.42996 | 0.20229 |
| 5 | N | N5 | 0.90859 | 0.63315 | 0.20272 |
| 6 | N | N6 | 0.13328 | 0.63318 | 0.20257 |
| 7 | N | N7 | 0.90855 | 0.40849 | 0.20279 |
| 8 | N | N8 | 0.13323 | 0.40851 | 0.20255 |
| 9 | N | N9 | 0.52094 | 0.61179 | 0.20137 |
| 10 | N | N10 | 0.43023 | 0.52081 | 0.20322 |
| 11 | N | N11 | 0.6116 | 0.52085 | 0.20321 |
| 12 | N | N12 | 0.5209 | 0.42986 | 0.20161 |
| 13 | N | N13 | 0.40862 | 0.63316 | 0.20216 |
| 14 | N | N14 | 0.63325 | 0.63321 | 0.20217 |
| 15 | N | N15 | 0.4086 | 0.40845 | 0.20241 |
| 16 | N | N16 | 0.63321 | 0.40848 | 0.20245 |
| 17 | N | N17 | 0.02098 | 0.11179 | 0.20152 |
| 18 | N | N18 | 0.93025 | 0.02082 | 0.2034 |
| 19 | N | N19 | 0.11162 | 0.02087 | 0.20358 |
| 20 | N | N20 | 0.02092 | 0.92989 | 0.20167 |
| 21 | N | N21 | 0.90866 | 0.13318 | 0.2022 |
| 22 | N | N22 | 0.13328 | 0.13322 | 0.20247 |
| 23 | N | N23 | 0.90862 | 0.90847 | 0.20236 |
| 24 | N | N24 | 0.13322 | 0.90849 | 0.2026 |
| 25 | N | N25 | 0.52096 | 0.11166 | 0.20207 |
| 26 | N | N26 | 0.43009 | 0.0208 | 0.20283 |
| 27 | N | N27 | 0.61178 | 0.02086 | 0.20277 |
| 28 | N | N28 | 0.52092 | 0.92999 | 0.20212 |
| 29 | N | N29 | 0.40858 | 0.13313 | 0.20241 |
| 30 | N | N30 | 0.63332 | 0.13318 | 0.20225 |
| 31 | N | N31 | 0.40856 | 0.90848 | 0.20238 |
| 32 | N | N32 | 0.63328 | 0.90851 | 0.20228 |
| 33 | N | N33 | 0.52093 | 0.52072 | 0.10727 |
| 34 | N | N34 | 0.02105 | 0.02079 | 0.10755 |
| 35 | N | N35 | 0.02084 | 0.52085 | 0.10798 |
| 36 | N | N36 | 0.52089 | 0.02084 | 0.10754 |
| 37 | C | C1 | 0.96837 | 0.65017 | 0.20253 |
| 38 | C | C2 | 0.89158 | 0.5734 | 0.20396 |
| 39 | C | C3 | 0.07348 | 0.6502 | 0.20247 |
| 40 | C | C4 | 0.89156 | 0.46824 | 0.20397 |
| 41 | C | C5 | 0.98752 | 0.7155 | 0.20318 |
| 42 | C | C6 | 0.82621 | 0.55422 | 0.20574 |
| 43 | C | C7 | 0.0543 | 0.71552 | 0.20318 |
| 44 | C | C8 | 0.8262 | 0.48744 | 0.2057 |
| 45 | C | C9 | 0.15026 | 0.57342 | 0.20378 |
| 46 | C | C10 | 0.96834 | 0.39147 | 0.20263 |
| 47 | C | C11 | 0.15023 | 0.46826 | 0.20374 |
| 48 | C | C12 | 0.07344 | 0.39149 | 0.20252 |
| 49 | C | C13 | 0.21562 | 0.55421 | 0.20551 |
| 50 | C | C14 | 0.2156 | 0.48743 | 0.20545 |
| 51 | C | C15 | 0.95231 | 0.77079 | 0.20307 |
| 52 | C | C16 | 0.77086 | 0.58938 | 0.20574 |
| 53 | C | C17 | 0.0895 | 0.7708 | 0.20316 |
| 54 | C | C18 | 0.77085 | 0.45226 | 0.20567 |
| 55 | C | C19 | 0.9875 | 0.32615 | 0.20324 |
| 56 | C | C20 | 0.05428 | 0.32618 | 0.20321 |
| 57 | C | C21 | 0.46839 | 0.65025 | 0.20188 |
| 58 | C | C22 | 0.39168 | 0.57339 | 0.20358 |
| 59 | C | C23 | 0.57347 | 0.65028 | 0.20188 |
| 60 | C | C24 | 0.39166 | 0.46822 | 0.20367 |
| 61 | C | C25 | 0.48752 | 0.7156 | 0.20274 |
| 62 | C | C26 | 0.32633 | 0.5542 | 0.20541 |
| 63 | C | C27 | 0.55431 | 0.71562 | 0.20274 |
| 64 | C | C28 | 0.32631 | 0.48741 | 0.2054 |
| 65 | C | C29 | 0.65017 | 0.57343 | 0.20359 |
| 66 | C | C30 | 0.46837 | 0.39137 | 0.20218 |
| 67 | C | C31 | 0.65014 | 0.46825 | 0.2037 |
| 68 | C | C32 | 0.57344 | 0.39139 | 0.2022 |
| 69 | C | C33 | 0.71551 | 0.55422 | 0.2055 |
| 70 | C | C34 | 0.71549 | 0.48743 | 0.2055 |
| 71 | C | C35 | 0.45232 | 0.77088 | 0.20294 |
| 72 | C | C36 | 0.27097 | 0.58937 | 0.20563 |
| 73 | C | C37 | 0.5895 | 0.7709 | 0.20293 |
| 74 | C | C38 | 0.27095 | 0.45225 | 0.20551 |
| 75 | C | C39 | 0.48751 | 0.32603 | 0.20306 |
| 76 | C | C40 | 0.5543 | 0.32605 | 0.20306 |
| 77 | C | C41 | 0.96843 | 0.15024 | 0.20194 |
| 78 | C | C42 | 0.89171 | 0.07341 | 0.20368 |
| 79 | C | C43 | 0.07351 | 0.15029 | 0.20206 |
| 80 | C | C44 | 0.89169 | 0.96824 | 0.20373 |
| 81 | C | C45 | 0.98755 | 0.21558 | 0.20282 |
| 82 | C | C46 | 0.82636 | 0.05421 | 0.20541 |
| 83 | C | C47 | 0.05433 | 0.21561 | 0.20286 |
| 84 | C | C48 | 0.82634 | 0.98744 | 0.20539 |
| 85 | C | C49 | 0.1502 | 0.07345 | 0.20394 |
| 86 | C | C50 | 0.96838 | 0.8914 | 0.20212 |
| 87 | C | C51 | 0.15015 | 0.96826 | 0.20395 |
| 88 | C | C52 | 0.07345 | 0.89142 | 0.20226 |
| 89 | C | C53 | 0.21554 | 0.05421 | 0.20571 |
| 90 | C | C54 | 0.21551 | 0.98744 | 0.20565 |
| 91 | C | C55 | 0.95232 | 0.27085 | 0.20304 |
| 92 | C | C56 | 0.771 | 0.08939 | 0.20553 |
| 93 | C | C57 | 0.08951 | 0.27091 | 0.20307 |
| 94 | C | C58 | 0.77099 | 0.95226 | 0.20542 |
| 95 | C | C59 | 0.98752 | 0.82607 | 0.203 |
| 96 | C | C60 | 0.0543 | 0.82608 | 0.20307 |
| 97 | C | C61 | 0.46837 | 0.15013 | 0.20231 |
| 98 | C | C62 | 0.39159 | 0.07337 | 0.20353 |
| 99 | C | C63 | 0.57352 | 0.15017 | 0.20223 |
| 100 | C | C64 | 0.39156 | 0.96824 | 0.20348 |
| 101 | C | C65 | 0.48753 | 0.21545 | 0.20304 |
| 102 | C | C66 | 0.32622 | 0.05419 | 0.20549 |
| 103 | C | C67 | 0.55431 | 0.21547 | 0.20299 |
| 104 | C | C68 | 0.3262 | 0.98742 | 0.20542 |
| 105 | C | C69 | 0.65031 | 0.07342 | 0.20338 |
| 106 | C | C70 | 0.46835 | 0.89149 | 0.20232 |
| 107 | C | C71 | 0.65028 | 0.96827 | 0.20337 |
| 108 | C | C72 | 0.57349 | 0.89151 | 0.20229 |
| 109 | C | C73 | 0.71567 | 0.05422 | 0.20526 |
| 110 | C | C74 | 0.71565 | 0.98744 | 0.20522 |
| 111 | C | C75 | 0.45231 | 0.27075 | 0.20311 |
| 112 | C | C76 | 0.27089 | 0.08937 | 0.20578 |
| 113 | C | C77 | 0.5895 | 0.27077 | 0.20307 |
| 114 | C | C78 | 0.27086 | 0.95225 | 0.2056 |
| 115 | C | C79 | 0.48753 | 0.82618 | 0.20299 |
| 116 | C | C80 | 0.55431 | 0.82619 | 0.20298 |
| 117 | H | H1 | 0.77085 | 0.64064 | 0.20491 |
| 118 | H | H2 | 0.77087 | 0.401 | 0.20472 |
| 119 | H | H3 | 0.14076 | 0.77077 | 0.20243 |
| 120 | H | H4 | 0.90105 | 0.77079 | 0.20226 |
| 121 | H | H5 | 0.27096 | 0.64063 | 0.20492 |
| 122 | H | H6 | 0.27091 | 0.40098 | 0.20464 |
| 123 | H | H7 | 0.64076 | 0.77087 | 0.20233 |
| 124 | H | H8 | 0.40106 | 0.77087 | 0.20231 |
| 125 | H | H9 | 0.771 | 0.14065 | 0.20487 |
| 126 | H | H10 | 0.77097 | 0.901 | 0.20459 |
| 127 | H | H11 | 0.14077 | 0.27095 | 0.20237 |
| 128 | H | H12 | 0.90105 | 0.27081 | 0.2023 |
| 129 | H | H13 | 0.27089 | 0.14063 | 0.20502 |
| 130 | H | H14 | 0.27085 | 0.90098 | 0.20463 |
| 131 | H | H15 | 0.64076 | 0.27076 | 0.20238 |
| 132 | H | H16 | 0.40105 | 0.27075 | 0.20242 |
| 133 | Fe | Fe1 | 0.0209 | 0.52083 | 0.18683 |
| 134 | Fe | Fe2 | 0.52092 | 0.52081 | 0.18663 |
| 135 | Fe | Fe3 | 0.02096 | 0.02083 | 0.1867 |
| 136 | Fe | Fe4 | 0.52093 | 0.02083 | 0.18625 |

**DFT-Optimized FeN_5_-SD_1_ Atomic Coordinates**

| **Serial number** | **element** | **Element sequence number** | **x-axis space position** | **y-axis space position** | **z-axis space position** |
| --- | --- | --- | --- | --- | --- |
| 1 | N | N1 | 0.03033 | 0.6029 | 0.23311 |
| 2 | N | N2 | 0.11343 | 0.52083 | 0.19398 |
| 3 | N | N3 | 0.03036 | 0.4387 | 0.23309 |
| 4 | N | N4 | 0.13035 | 0.63386 | 0.19305 |
| 5 | N | N5 | 0.13042 | 0.40781 | 0.19311 |
| 6 | N | N6 | 0.52147 | 0.61142 | 0.20381 |
| 7 | N | N7 | 0.43012 | 0.52096 | 0.20012 |
| 8 | N | N8 | 0.61276 | 0.52104 | 0.20165 |
| 9 | N | N9 | 0.5215 | 0.43055 | 0.20382 |
| 10 | N | N10 | 0.40902 | 0.6333 | 0.2011 |
| 11 | N | N11 | 0.63397 | 0.63328 | 0.20283 |
| 12 | N | N12 | 0.40907 | 0.40861 | 0.20112 |
| 13 | N | N13 | 0.634 | 0.40879 | 0.20282 |
| 14 | N | N14 | 0.02181 | 0.11224 | 0.20054 |
| 15 | N | N15 | 0.9306 | 0.02083 | 0.2023 |
| 16 | N | N16 | 0.11152 | 0.02081 | 0.20551 |
| 17 | N | N17 | 0.02177 | 0.92938 | 0.20051 |
| 18 | N | N18 | 0.9089 | 0.13273 | 0.19807 |
| 19 | N | N19 | 0.13328 | 0.13392 | 0.20494 |
| 20 | N | N20 | 0.90884 | 0.90895 | 0.1982 |
| 21 | N | N21 | 0.13324 | 0.90768 | 0.20484 |
| 22 | N | N22 | 0.52105 | 0.11192 | 0.20235 |
| 23 | N | N23 | 0.43003 | 0.02097 | 0.20232 |
| 24 | N | N24 | 0.61196 | 0.02098 | 0.20203 |
| 25 | N | N25 | 0.52101 | 0.93003 | 0.20235 |
| 26 | N | N26 | 0.40865 | 0.13335 | 0.20302 |
| 27 | N | N27 | 0.63345 | 0.13334 | 0.20252 |
| 28 | N | N28 | 0.40863 | 0.90857 | 0.20301 |
| 29 | N | N29 | 0.63339 | 0.9086 | 0.20253 |
| 30 | N | N30 | 0.52195 | 0.52099 | 0.10734 |
| 31 | N | N31 | 0.02283 | 0.02086 | 0.10738 |
| 32 | N | N32 | 0.99216 | 0.52084 | 0.13202 |
| 33 | N | N33 | 0.52098 | 0.02099 | 0.10719 |
| 34 | C | C1 | 0.07068 | 0.64663 | 0.20859 |
| 35 | C | C2 | 0.97925 | 0.71615 | 0.19162 |
| 36 | C | C3 | 0.82883 | 0.55414 | 0.20139 |
| 37 | C | C4 | 0.04545 | 0.71105 | 0.20108 |
| 38 | C | C5 | 0.82881 | 0.48788 | 0.20139 |
| 39 | C | C6 | 0.15064 | 0.57435 | 0.19383 |
| 40 | C | C7 | 0.15066 | 0.46733 | 0.19385 |
| 41 | C | C8 | 0.07075 | 0.39498 | 0.20864 |
| 42 | C | C9 | 0.21564 | 0.55462 | 0.19403 |
| 43 | C | C10 | 0.21565 | 0.48709 | 0.19405 |
| 44 | C | C11 | 0.94909 | 0.7737 | 0.19067 |
| 45 | C | C12 | 0.77281 | 0.58826 | 0.20245 |
| 46 | C | C13 | 0.08304 | 0.7657 | 0.20455 |
| 47 | C | C14 | 0.77279 | 0.45378 | 0.20245 |
| 48 | C | C15 | 0.97938 | 0.32545 | 0.19153 |
| 49 | C | C16 | 0.04557 | 0.33053 | 0.20111 |
| 50 | C | C17 | 0.46872 | 0.64996 | 0.20372 |
| 51 | C | C18 | 0.39162 | 0.57348 | 0.19973 |
| 52 | C | C19 | 0.57412 | 0.65009 | 0.20428 |
| 53 | C | C20 | 0.39165 | 0.46842 | 0.19974 |
| 54 | C | C21 | 0.48799 | 0.71535 | 0.2057 |
| 55 | C | C22 | 0.32647 | 0.55437 | 0.19814 |
| 56 | C | C23 | 0.55476 | 0.7154 | 0.2059 |
| 57 | C | C24 | 0.32649 | 0.48746 | 0.19814 |
| 58 | C | C25 | 0.65123 | 0.57354 | 0.20224 |
| 59 | C | C26 | 0.46878 | 0.39198 | 0.20374 |
| 60 | C | C27 | 0.65122 | 0.46853 | 0.20224 |
| 61 | C | C28 | 0.57417 | 0.39191 | 0.20427 |
| 62 | C | C29 | 0.7167 | 0.55413 | 0.20274 |
| 63 | C | C30 | 0.71669 | 0.48794 | 0.20274 |
| 64 | C | C31 | 0.4527 | 0.77062 | 0.20569 |
| 65 | C | C32 | 0.2711 | 0.58943 | 0.19634 |
| 66 | C | C33 | 0.58974 | 0.7709 | 0.20585 |
| 67 | C | C34 | 0.27116 | 0.45234 | 0.19636 |
| 68 | C | C35 | 0.48807 | 0.3266 | 0.20572 |
| 69 | C | C36 | 0.55484 | 0.32659 | 0.20589 |
| 70 | C | C37 | 0.96882 | 0.14956 | 0.19798 |
| 71 | C | C38 | 0.89191 | 0.07327 | 0.20168 |
| 72 | C | C39 | 0.07376 | 0.15169 | 0.20297 |
| 73 | C | C40 | 0.89187 | 0.96842 | 0.20173 |
| 74 | C | C41 | 0.98664 | 0.21461 | 0.19707 |
| 75 | C | C42 | 0.82651 | 0.05421 | 0.20381 |
| 76 | C | C43 | 0.05279 | 0.21755 | 0.20184 |
| 77 | C | C44 | 0.82648 | 0.98751 | 0.20383 |
| 78 | C | C45 | 0.14998 | 0.07375 | 0.20598 |
| 79 | C | C46 | 0.96875 | 0.89207 | 0.19805 |
| 80 | C | C47 | 0.14997 | 0.96784 | 0.20592 |
| 81 | C | C48 | 0.07372 | 0.8899 | 0.20288 |
| 82 | C | C49 | 0.21523 | 0.05425 | 0.20719 |
| 83 | C | C50 | 0.21524 | 0.98735 | 0.20717 |
| 84 | C | C51 | 0.9492 | 0.26792 | 0.19055 |
| 85 | C | C52 | 0.77121 | 0.08942 | 0.20432 |
| 86 | C | C53 | 0.08315 | 0.27587 | 0.20464 |
| 87 | C | C54 | 0.77114 | 0.95235 | 0.20436 |
| 88 | C | C55 | 0.98655 | 0.827 | 0.19714 |
| 89 | C | C56 | 0.0527 | 0.82404 | 0.2018 |
| 90 | C | C57 | 0.46846 | 0.15045 | 0.20329 |
| 91 | C | C58 | 0.39155 | 0.07359 | 0.20344 |
| 92 | C | C59 | 0.57368 | 0.1504 | 0.20315 |
| 93 | C | C60 | 0.39154 | 0.96833 | 0.20345 |
| 94 | C | C61 | 0.48773 | 0.21583 | 0.20511 |
| 95 | C | C62 | 0.32612 | 0.05434 | 0.20568 |
| 96 | C | C63 | 0.55454 | 0.2158 | 0.20512 |
| 97 | C | C64 | 0.32613 | 0.98752 | 0.20568 |
| 98 | C | C65 | 0.65048 | 0.07355 | 0.20277 |
| 99 | C | C66 | 0.46843 | 0.89147 | 0.2033 |
| 100 | C | C67 | 0.65042 | 0.96838 | 0.20278 |
| 101 | C | C68 | 0.57362 | 0.89156 | 0.20316 |
| 102 | C | C69 | 0.71583 | 0.05432 | 0.20418 |
| 103 | C | C70 | 0.7158 | 0.98753 | 0.20419 |
| 104 | C | C71 | 0.45278 | 0.27134 | 0.2057 |
| 105 | C | C72 | 0.27071 | 0.08936 | 0.20676 |
| 106 | C | C73 | 0.58981 | 0.27106 | 0.20584 |
| 107 | C | C74 | 0.2708 | 0.95236 | 0.20675 |
| 108 | C | C75 | 0.48768 | 0.82611 | 0.20512 |
| 109 | C | C76 | 0.55448 | 0.82617 | 0.20512 |
| 110 | H | H1 | 0.77227 | 0.63956 | 0.20241 |
| 111 | H | H2 | 0.77221 | 0.40247 | 0.2024 |
| 112 | H | H3 | 0.13391 | 0.76162 | 0.21017 |
| 113 | H | H4 | 0.89839 | 0.77807 | 0.18392 |
| 114 | H | H5 | 0.27125 | 0.64071 | 0.19665 |
| 115 | H | H6 | 0.27137 | 0.40106 | 0.19671 |
| 116 | H | H7 | 0.64101 | 0.77104 | 0.20552 |
| 117 | H | H8 | 0.40144 | 0.77044 | 0.20524 |
| 118 | H | H9 | 0.77115 | 0.14068 | 0.20413 |
| 119 | H | H10 | 0.77103 | 0.90109 | 0.20418 |
| 120 | H | H11 | 0.13401 | 0.27994 | 0.21034 |
| 121 | H | H12 | 0.89851 | 0.26354 | 0.18372 |
| 122 | H | H13 | 0.27066 | 0.14062 | 0.20642 |
| 123 | H | H14 | 0.27091 | 0.9011 | 0.20641 |
| 124 | H | H15 | 0.64108 | 0.27087 | 0.20551 |
| 125 | H | H16 | 0.40152 | 0.27153 | 0.20526 |
| 126 | H | H17 | 0.95183 | 0.36791 | 0.18253 |
| 127 | H | H18 | 0.87388 | 0.5787 | 0.19998 |
| 128 | H | H19 | 0.87386 | 0.46328 | 0.19997 |
| 129 | H | H20 | 0.95167 | 0.6737 | 0.18269 |
| 130 | H | H21 | 0.98987 | 0.62216 | 0.25118 |
| 131 | H | H22 | 0.96512 | 0.52078 | 0.24162 |
| 132 | H | H23 | 0.9899 | 0.41941 | 0.25113 |
| 133 | Fe | Fe1 | 0.02402 | 0.52081 | 0.20033 |
| 134 | Fe | Fe2 | 0.52157 | 0.52099 | 0.18633 |
| 135 | Fe | Fe3 | 0.02182 | 0.02082 | 0.18652 |
| 136 | Fe | Fe4 | 0.52096 | 0.02098 | 0.18638 |

**DFT-Optimized FeN_5_-SD_2_ Atomic Coordinates**

| **Serial number** | **element** | **Element sequence number** | **x-axis space position** | **y-axis space position** | **z-axis space position** |
| --- | --- | --- | --- | --- | --- |
| 1 | N | N1 | 0.52095 | 0.61192 | 0.20086 |
| 2 | N | N2 | 0.43031 | 0.52079 | 0.20401 |
| 3 | N | N3 | 0.61145 | 0.52082 | 0.20407 |
| 4 | N | N4 | 0.52084 | 0.42967 | 0.20111 |
| 5 | N | N5 | 0.40856 | 0.63307 | 0.20227 |
| 6 | N | N6 | 0.63332 | 0.63308 | 0.20227 |
| 7 | N | N7 | 0.40851 | 0.40849 | 0.20242 |
| 8 | N | N8 | 0.63317 | 0.40849 | 0.20247 |
| 9 | N | N9 | 0.02086 | 0.11154 | 0.20377 |
| 10 | N | N10 | 0.92975 | 0.02057 | 0.2016 |
| 11 | N | N11 | 0.11204 | 0.02117 | 0.2018 |
| 12 | N | N12 | 0.02095 | 0.93017 | 0.20393 |
| 13 | N | N13 | 0.90845 | 0.13286 | 0.20248 |
| 14 | N | N14 | 0.13308 | 0.13359 | 0.20264 |
| 15 | N | N15 | 0.90869 | 0.9082 | 0.20253 |
| 16 | N | N16 | 0.1333 | 0.90881 | 0.20274 |
| 17 | N | N17 | 0.52098 | 0.11169 | 0.20164 |
| 18 | N | N18 | 0.43023 | 0.02085 | 0.20293 |
| 19 | N | N19 | 0.61157 | 0.02081 | 0.20285 |
| 20 | N | N20 | 0.52085 | 0.92995 | 0.20167 |
| 21 | N | N21 | 0.40864 | 0.13321 | 0.20232 |
| 22 | N | N22 | 0.63332 | 0.13317 | 0.20221 |
| 23 | N | N23 | 0.4085 | 0.9085 | 0.20228 |
| 24 | N | N24 | 0.63318 | 0.90843 | 0.20221 |
| 25 | N | N25 | 0.52092 | 0.52066 | 0.10752 |
| 26 | N | N26 | 0.02107 | 0.02076 | 0.10786 |
| 27 | N | N27 | 0.52087 | 0.0208 | 0.10728 |
| 28 | C | C1 | 0.98857 | 0.71375 | 0.20107 |
| 29 | C | C2 | 0.82783 | 0.55341 | 0.20447 |
| 30 | C | C3 | 0.05488 | 0.71402 | 0.20115 |
| 31 | C | C4 | 0.82763 | 0.4871 | 0.20443 |
| 32 | C | C5 | 0.21398 | 0.55367 | 0.20458 |
| 33 | C | C6 | 0.21406 | 0.48737 | 0.20451 |
| 34 | C | C7 | 0.95426 | 0.76967 | 0.20199 |
| 35 | C | C8 | 0.77188 | 0.58766 | 0.20475 |
| 36 | C | C9 | 0.08869 | 0.77021 | 0.20215 |
| 37 | C | C10 | 0.77147 | 0.45323 | 0.20473 |
| 38 | C | C11 | 0.98601 | 0.32752 | 0.20111 |
| 39 | C | C12 | 0.05232 | 0.32809 | 0.20115 |
| 40 | C | C13 | 0.46842 | 0.6503 | 0.20179 |
| 41 | C | C14 | 0.39167 | 0.5734 | 0.20371 |
| 42 | C | C15 | 0.57345 | 0.65032 | 0.20179 |
| 43 | C | C16 | 0.39169 | 0.46819 | 0.20376 |
| 44 | C | C17 | 0.4875 | 0.71561 | 0.20287 |
| 45 | C | C18 | 0.3262 | 0.55381 | 0.20474 |
| 46 | C | C19 | 0.55432 | 0.71563 | 0.20286 |
| 47 | C | C20 | 0.32625 | 0.48768 | 0.20473 |
| 48 | C | C21 | 0.65016 | 0.57338 | 0.20374 |
| 49 | C | C22 | 0.46836 | 0.39126 | 0.20202 |
| 50 | C | C23 | 0.65 | 0.46817 | 0.20383 |
| 51 | C | C24 | 0.57332 | 0.39128 | 0.20204 |
| 52 | C | C25 | 0.71561 | 0.55372 | 0.20476 |
| 53 | C | C26 | 0.71546 | 0.48759 | 0.20479 |
| 54 | C | C27 | 0.45229 | 0.77087 | 0.2032 |
| 55 | C | C28 | 0.26997 | 0.58784 | 0.20482 |
| 56 | C | C29 | 0.58946 | 0.77092 | 0.2032 |
| 57 | C | C30 | 0.27018 | 0.45342 | 0.20472 |
| 58 | C | C31 | 0.48744 | 0.32596 | 0.2031 |
| 59 | C | C32 | 0.55425 | 0.32599 | 0.2031 |
| 60 | C | C33 | 0.96811 | 0.14987 | 0.20295 |
| 61 | C | C34 | 0.89132 | 0.07301 | 0.20299 |
| 62 | C | C35 | 0.07333 | 0.1504 | 0.20301 |
| 63 | C | C36 | 0.89137 | 0.96802 | 0.20298 |
| 64 | C | C37 | 0.98727 | 0.21538 | 0.20271 |
| 65 | C | C38 | 0.82602 | 0.05392 | 0.2051 |
| 66 | C | C39 | 0.05341 | 0.21582 | 0.20274 |
| 67 | C | C40 | 0.826 | 0.9871 | 0.20506 |
| 68 | C | C41 | 0.15041 | 0.07374 | 0.20319 |
| 69 | C | C42 | 0.96843 | 0.89141 | 0.20308 |
| 70 | C | C43 | 0.15044 | 0.96868 | 0.20321 |
| 71 | C | C44 | 0.07365 | 0.89174 | 0.20319 |
| 72 | C | C45 | 0.21577 | 0.0546 | 0.20535 |
| 73 | C | C46 | 0.21575 | 0.98777 | 0.20531 |
| 74 | C | C47 | 0.95254 | 0.27114 | 0.20197 |
| 75 | C | C48 | 0.7708 | 0.08918 | 0.20555 |
| 76 | C | C49 | 0.08695 | 0.27236 | 0.20205 |
| 77 | C | C50 | 0.77065 | 0.95203 | 0.20545 |
| 78 | C | C51 | 0.98816 | 0.82598 | 0.20285 |
| 79 | C | C52 | 0.05431 | 0.82622 | 0.20292 |
| 80 | C | C53 | 0.46844 | 0.15017 | 0.20218 |
| 81 | C | C54 | 0.39171 | 0.07345 | 0.20353 |
| 82 | C | C55 | 0.57353 | 0.15019 | 0.20213 |
| 83 | C | C56 | 0.39163 | 0.96828 | 0.2035 |
| 84 | C | C57 | 0.48757 | 0.21546 | 0.20314 |
| 85 | C | C58 | 0.32641 | 0.05432 | 0.20544 |
| 86 | C | C59 | 0.55433 | 0.21548 | 0.20311 |
| 87 | C | C60 | 0.32636 | 0.98757 | 0.20539 |
| 88 | C | C61 | 0.65017 | 0.07338 | 0.20339 |
| 89 | C | C62 | 0.46829 | 0.89146 | 0.20216 |
| 90 | C | C63 | 0.65009 | 0.9682 | 0.20339 |
| 91 | C | C64 | 0.57338 | 0.89145 | 0.20215 |
| 92 | C | C65 | 0.71544 | 0.05408 | 0.20524 |
| 93 | C | C66 | 0.71539 | 0.98733 | 0.20521 |
| 94 | C | C67 | 0.4523 | 0.27068 | 0.20335 |
| 95 | C | C68 | 0.27115 | 0.08963 | 0.20575 |
| 96 | C | C69 | 0.58948 | 0.27076 | 0.20333 |
| 97 | C | C70 | 0.27097 | 0.9525 | 0.20565 |
| 98 | C | C71 | 0.48746 | 0.82615 | 0.20306 |
| 99 | C | C72 | 0.55423 | 0.82616 | 0.20307 |
| 100 | H | H1 | 0.77159 | 0.63896 | 0.2046 |
| 101 | H | H2 | 0.77073 | 0.40193 | 0.20452 |
| 102 | H | H3 | 0.13998 | 0.77096 | 0.20201 |
| 103 | H | H4 | 0.90296 | 0.7699 | 0.20173 |
| 104 | H | H5 | 0.27033 | 0.63915 | 0.20469 |
| 105 | H | H6 | 0.2708 | 0.40212 | 0.20448 |
| 106 | H | H7 | 0.64071 | 0.77096 | 0.20281 |
| 107 | H | H8 | 0.40104 | 0.77085 | 0.20279 |
| 108 | H | H9 | 0.77091 | 0.14043 | 0.20511 |
| 109 | H | H10 | 0.77052 | 0.90078 | 0.20492 |
| 110 | H | H11 | 0.13826 | 0.2725 | 0.2019 |
| 111 | H | H12 | 0.90125 | 0.27 | 0.20176 |
| 112 | H | H13 | 0.2713 | 0.14088 | 0.20529 |
| 113 | H | H14 | 0.27084 | 0.90124 | 0.20506 |
| 114 | H | H15 | 0.64074 | 0.27079 | 0.2029 |
| 115 | H | H16 | 0.40104 | 0.27059 | 0.20293 |
| 116 | H | H17 | 0.16907 | 0.5786 | 0.20429 |
| 117 | H | H18 | 0.16923 | 0.4623 | 0.20411 |
| 118 | H | H19 | 0.87278 | 0.57825 | 0.20412 |
| 119 | H | H20 | 0.87241 | 0.46194 | 0.20401 |
| 120 | H | H21 | 0.08008 | 0.66927 | 0.20024 |
| 121 | H | H22 | 0.07687 | 0.37321 | 0.20031 |
| 122 | H | H23 | 0.96056 | 0.37213 | 0.20024 |
| 123 | H | H24 | 0.96377 | 0.66878 | 0.20011 |
| 124 | Fe | Fe1 | 0.52088 | 0.5208 | 0.1867 |
| 125 | Fe | Fe2 | 0.02094 | 0.02084 | 0.18704 |
| 126 | Fe | Fe3 | 0.5209 | 0.02082 | 0.18631 |

**DFT-Optimized FeN_5_-SD_3_ Atomic Coordinates**

| **Serial number** | **element** | **Element sequence number** | **x-axis space position** | **y-axis space position** | **z-axis space position** |
| --- | --- | --- | --- | --- | --- |
| 1 | N | N1 | 0.52113 | 0.61264 | 0.20097 |
| 2 | N | N2 | 0.43073 | 0.52126 | 0.20405 |
| 3 | N | N3 | 0.61172 | 0.52148 | 0.20382 |
| 4 | N | N4 | 0.52137 | 0.43005 | 0.19995 |
| 5 | N | N5 | 0.40871 | 0.63354 | 0.20213 |
| 6 | N | N6 | 0.63343 | 0.63388 | 0.20191 |
| 7 | N | N7 | 0.40896 | 0.40895 | 0.20122 |
| 8 | N | N8 | 0.63379 | 0.40914 | 0.2008 |
| 9 | N | N9 | 0.02066 | 0.11164 | 0.20369 |
| 10 | N | N10 | 0.92975 | 0.02109 | 0.20358 |
| 11 | N | N11 | 0.11158 | 0.02102 | 0.20347 |
| 12 | N | N12 | 0.02068 | 0.9291 | 0.19969 |
| 13 | N | N13 | 0.90794 | 0.133 | 0.20515 |
| 14 | N | N14 | 0.13337 | 0.13291 | 0.20538 |
| 15 | N | N15 | 0.90898 | 0.90802 | 0.19815 |
| 16 | N | N16 | 0.13237 | 0.90794 | 0.19766 |
| 17 | N | N17 | 0.52072 | 0.11356 | 0.19274 |
| 18 | N | N18 | 0.43764 | 0.03251 | 0.23575 |
| 19 | N | N19 | 0.60351 | 0.03209 | 0.2353 |
| 20 | N | N20 | 0.40758 | 0.13095 | 0.19107 |
| 21 | N | N21 | 0.63392 | 0.1305 | 0.19075 |
| 22 | N | N22 | 0.52115 | 0.52147 | 0.10728 |
| 23 | N | N23 | 0.02063 | 0.02272 | 0.10807 |
| 24 | N | N24 | 0.52024 | 0.98817 | 0.13886 |
| 25 | C | C1 | 0.98722 | 0.71269 | 0.20252 |
| 26 | C | C2 | 0.82804 | 0.55469 | 0.20607 |
| 27 | C | C3 | 0.05345 | 0.71258 | 0.20256 |
| 28 | C | C4 | 0.82806 | 0.48841 | 0.20662 |
| 29 | C | C5 | 0.21426 | 0.55363 | 0.2066 |
| 30 | C | C6 | 0.21451 | 0.48735 | 0.20721 |
| 31 | C | C7 | 0.95328 | 0.76889 | 0.20241 |
| 32 | C | C8 | 0.77197 | 0.58874 | 0.20549 |
| 33 | C | C9 | 0.08763 | 0.76865 | 0.20243 |
| 34 | C | C10 | 0.77197 | 0.45436 | 0.20637 |
| 35 | C | C11 | 0.98786 | 0.32785 | 0.2038 |
| 36 | C | C12 | 0.0541 | 0.32774 | 0.20379 |
| 37 | C | C13 | 0.46856 | 0.65095 | 0.20147 |
| 38 | C | C14 | 0.39195 | 0.57385 | 0.20392 |
| 39 | C | C15 | 0.57354 | 0.65117 | 0.20135 |
| 40 | C | C16 | 0.39223 | 0.46858 | 0.20402 |
| 41 | C | C17 | 0.48779 | 0.71633 | 0.20157 |
| 42 | C | C18 | 0.32651 | 0.55409 | 0.20559 |
| 43 | C | C19 | 0.55401 | 0.7165 | 0.20147 |
| 44 | C | C20 | 0.32671 | 0.48798 | 0.20595 |
| 45 | C | C21 | 0.65032 | 0.5742 | 0.20367 |
| 46 | C | C22 | 0.46888 | 0.39164 | 0.19937 |
| 47 | C | C23 | 0.65036 | 0.46885 | 0.20362 |
| 48 | C | C24 | 0.57391 | 0.39164 | 0.19913 |
| 49 | C | C25 | 0.7158 | 0.55463 | 0.20522 |
| 50 | C | C26 | 0.71583 | 0.4885 | 0.20547 |
| 51 | C | C27 | 0.45346 | 0.77232 | 0.20062 |
| 52 | C | C28 | 0.27018 | 0.58794 | 0.20591 |
| 53 | C | C29 | 0.58792 | 0.77275 | 0.20039 |
| 54 | C | C30 | 0.27076 | 0.45357 | 0.20698 |
| 55 | C | C31 | 0.48788 | 0.32654 | 0.19677 |
| 56 | C | C32 | 0.55483 | 0.32648 | 0.19659 |
| 57 | C | C33 | 0.96791 | 0.15004 | 0.20485 |
| 58 | C | C34 | 0.89034 | 0.07329 | 0.2047 |
| 59 | C | C35 | 0.0734 | 0.14997 | 0.20496 |
| 60 | C | C36 | 0.89232 | 0.96808 | 0.19955 |
| 61 | C | C37 | 0.98761 | 0.21557 | 0.2057 |
| 62 | C | C38 | 0.8246 | 0.05245 | 0.20287 |
| 63 | C | C39 | 0.05382 | 0.21551 | 0.20575 |
| 64 | C | C40 | 0.82731 | 0.98627 | 0.19789 |
| 65 | C | C41 | 0.15093 | 0.0732 | 0.20479 |
| 66 | C | C42 | 0.96838 | 0.89058 | 0.20004 |
| 67 | C | C43 | 0.14906 | 0.96801 | 0.19915 |
| 68 | C | C44 | 0.07296 | 0.89049 | 0.19982 |
| 69 | C | C45 | 0.21666 | 0.05247 | 0.20291 |
| 70 | C | C46 | 0.21406 | 0.98632 | 0.19748 |
| 71 | C | C47 | 0.95368 | 0.27181 | 0.20504 |
| 72 | C | C48 | 0.7663 | 0.08319 | 0.20503 |
| 73 | C | C49 | 0.08802 | 0.27156 | 0.20505 |
| 74 | C | C50 | 0.77362 | 0.94937 | 0.19026 |
| 75 | C | C51 | 0.98755 | 0.82488 | 0.20174 |
| 76 | C | C52 | 0.05364 | 0.82481 | 0.20167 |
| 77 | C | C53 | 0.46731 | 0.15099 | 0.19172 |
| 78 | C | C54 | 0.39437 | 0.07205 | 0.20881 |
| 79 | C | C55 | 0.5743 | 0.15078 | 0.19148 |
| 80 | C | C56 | 0.48722 | 0.21585 | 0.19118 |
| 81 | C | C57 | 0.3298 | 0.04656 | 0.20114 |
| 82 | C | C58 | 0.55472 | 0.21572 | 0.191 |
| 83 | C | C59 | 0.32517 | 0.98044 | 0.19069 |
| 84 | C | C60 | 0.64691 | 0.07155 | 0.20849 |
| 85 | C | C61 | 0.71147 | 0.04609 | 0.20106 |
| 86 | C | C62 | 0.71626 | 0.97992 | 0.19114 |
| 87 | C | C63 | 0.45258 | 0.27143 | 0.19418 |
| 88 | C | C64 | 0.27483 | 0.08343 | 0.20535 |
| 89 | C | C65 | 0.58972 | 0.27111 | 0.19379 |
| 90 | C | C66 | 0.2679 | 0.94968 | 0.18958 |
| 91 | C | C67 | 0.48733 | 0.82844 | 0.19882 |
| 92 | C | C68 | 0.55364 | 0.82864 | 0.1987 |
| 93 | H | H1 | 0.77152 | 0.64003 | 0.20497 |
| 94 | H | H2 | 0.77149 | 0.40305 | 0.20663 |
| 95 | H | H3 | 0.13894 | 0.76899 | 0.20247 |
| 96 | H | H4 | 0.90198 | 0.7695 | 0.20243 |
| 97 | H | H5 | 0.27039 | 0.63924 | 0.20532 |
| 98 | H | H6 | 0.27149 | 0.40226 | 0.2073 |
| 99 | H | H7 | 0.63923 | 0.77234 | 0.20007 |
| 100 | H | H8 | 0.40215 | 0.7715 | 0.20044 |
| 101 | H | H9 | 0.76261 | 0.13408 | 0.21072 |
| 102 | H | H10 | 0.7775 | 0.89871 | 0.18289 |
| 103 | H | H11 | 0.13932 | 0.27091 | 0.20476 |
| 104 | H | H12 | 0.90237 | 0.27148 | 0.20474 |
| 105 | H | H13 | 0.27824 | 0.13429 | 0.21149 |
| 106 | H | H14 | 0.26425 | 0.89906 | 0.18175 |
| 107 | H | H15 | 0.64101 | 0.27107 | 0.19395 |
| 108 | H | H16 | 0.40131 | 0.27182 | 0.19466 |
| 109 | H | H17 | 0.46259 | 0.87339 | 0.19683 |
| 110 | H | H18 | 0.57807 | 0.87375 | 0.19665 |
| 111 | H | H19 | 0.87292 | 0.57968 | 0.20602 |
| 112 | H | H20 | 0.87293 | 0.46339 | 0.2071 |
| 113 | H | H21 | 0.96211 | 0.6679 | 0.20235 |
| 114 | H | H22 | 0.96292 | 0.37273 | 0.20243 |
| 115 | H | H23 | 0.36801 | 0.95356 | 0.18127 |
| 116 | H | H24 | 0.41796 | 0.99283 | 0.25519 |
| 117 | H | H25 | 0.52042 | 0.96887 | 0.25067 |
| 118 | H | H26 | 0.62304 | 0.99231 | 0.25471 |
| 119 | H | H27 | 0.67351 | 0.95283 | 0.18192 |
| 120 | H | H28 | 0.07839 | 0.66769 | 0.20246 |
| 121 | H | H29 | 0.16929 | 0.57845 | 0.20653 |
| 122 | H | H30 | 0.16974 | 0.46215 | 0.20775 |
| 123 | H | H31 | 0.07923 | 0.37252 | 0.2024 |
| 124 | Fe | Fe1 | 0.52123 | 0.52142 | 0.18636 |
| 125 | Fe | Fe2 | 0.02066 | 0.02126 | 0.18742 |
| 126 | Fe | Fe3 | 0.52049 | 0.02454 | 0.20453 |
